# The diversity of three-dimensional photonic crystals

**DOI:** 10.1038/s41467-021-22809-6

**Published:** 2021-05-05

**Authors:** Rose K. Cersonsky, James Antonaglia, Bradley D. Dice, Sharon C. Glotzer

**Affiliations:** 1 Macromolecular Science and Engineering Program, University of Michigan, Ann Arbor, MI USA; 2 Department of Physics, University of Michigan, Ann Arbor, MI USA; 3 Department of Chemical Engineering, University of Michigan, Ann Arbor, MI USA; 4 Department of Materials Science and Engineering, University of Michigan, Ann Arbor, MI USA; 5 Biointerfaces Institute, University of Michigan, Ann Arbor, MI USA

**Keywords:** Photonic crystals, Colloids, Theory and computation, Photonic crystals

## Abstract

Many butterflies, birds, beetles, and chameleons owe their spectacular colors to the microscopic patterns within their wings, feathers, or skin. When these patterns, or photonic crystals, result in the omnidirectional reflection of commensurate wavelengths of light, it is due to a complete photonic band gap (PBG). The number of natural crystal structures known to have a PBG is relatively small, and those within the even smaller subset of notoriety, including diamond and inverse opal, have proven difficult to synthesize. Here, we report more than 150,000 photonic band calculations for thousands of natural crystal templates from which we predict 351 photonic crystal templates – including nearly 300 previously-unreported structures – that can potentially be realized for a multitude of applications and length scales, including several in the visible range via colloidal self-assembly. With this large variety of 3D photonic crystals, we also revisit and discuss oft-used primary design heuristics for PBG materials.

## Abstract (web summary)

Photonic crystals have a range of desirable properties for manipulating light. Here, the authors calculate and use the photonic band gap for thousands of such crystals to examine heuristics for their design and predict new photonic crystal structures.

## Introduction

When will a 3D crystal possess an omnidirectional photonic band gap (PBG)? By definition, when there exists a range of frequencies which are not transmittable through the crystal due to the interactions of waves moving through media of different permittivity. For a material whose spatially dependent dielectric constant is given by *ε*(**r**), the transmittable frequencies are given by the eigenvalues of

1 $$\nabla \times \frac{1}{\varepsilon ({\bf{r}})}\nabla \times {\bf{H}}({\bf{r}})={\left(\frac{\omega }{c}\right)}^{2}{\bf{H}}({\bf{r}}),$$

as derived from Maxwell’s equations and solved in reciprocal space, where **H**(**r**) is the magnetic field, *ω* is the frequency, and *c* is the speed of light. Before the introduction of efficient computational packages such as MPB, MEEP, or COMSOL^1–3^, solving this equation was mathematically burdensome, thus the photonics community developed several heuristics to guide the design of photonic crystals, both from the more straightforward 2D and select 3D photonic crystals.

Yablonovitch^4^, John^5^, and Ho, et al.^6^ pointed to the sphericity of the Brillouin Zone (BZ), which is the reciprocal space analog to the real space primitive unit cell, as an important feature for predicting which photonic crystals have a PBG. They reasoned that cubic face-centered (*cF*) and body-centered (*cI*) lattices are the most likely targets for PBGs, as they have the most spherical BZs.

The study of 2D PBGs has also provided useful insight into photonic crystal design. Meade, et al. explain the origins of PBGs in 2D crystals by looking at the modes of the photonic band structure defining the PBG. They showed that PBGs occur when there is a large shift in where the electric energy density is localized, where the electric energy density is given by integrating the product of the electric field **E**(**r**) and displacement field **D**(**r**) = *ε*(**r**)**E**(**r**). In the band below the PBG, the electric energy density primarily resides in the medium with the higher dielectric constant (hereon “dielectric”); this band is known as the “dielectric band” where dielectric constant *ε* is the square of the index of refraction *n*. Above the PBG, the electric energy density is in the medium with the lower dielectric constant (hereon "air”); this band is known as the “air band.” Meade, et al. suggest one must aim to decrease the energy of the dielectric band in order to increase the size of the PBG. For a PBG between the transverse electric (TE) modes, this requires that regions of dielectric be connected^7^. Likewise, the importance of energy localization suggests that the greater the difference in *ε* of the two regions, the larger the PBG, as this will decrease the similarity between the dielectric and air band. These 2D principles are used to understand and design 3D photonic crystals, the terminology of electric and air band has become conventional in 3D PBG crystals.^8^

In this work, we revisit these heuristics—connectivity of the dielectric, increasing gap size with increasing *ε*, and spherical BZ—given a large dataset of 3D photonic band structure calculations, 151,593 in total, and discuss, despite their usefulness, the inadvertent restrictions that they may have imposed on the design search space of PBG crystals to structures near diamond and inverse opal, both of which are face-centered cubic structures that exhibit PBGs when the dielectric forms a continuous network^6,8–17^. From our calculations, we demonstrate that an omnidirectional PBG can be supported in all Bravais lattices, in systems of connected and disconnected high dielectric media, and that in some systems, the largest band gap is possible with an intermediate dielectric constant. Furthermore, we provide a comprehensive dataset of PBG crystal possibilities, opening multiple avenues for further study.

## Results

**Fig. 1:**
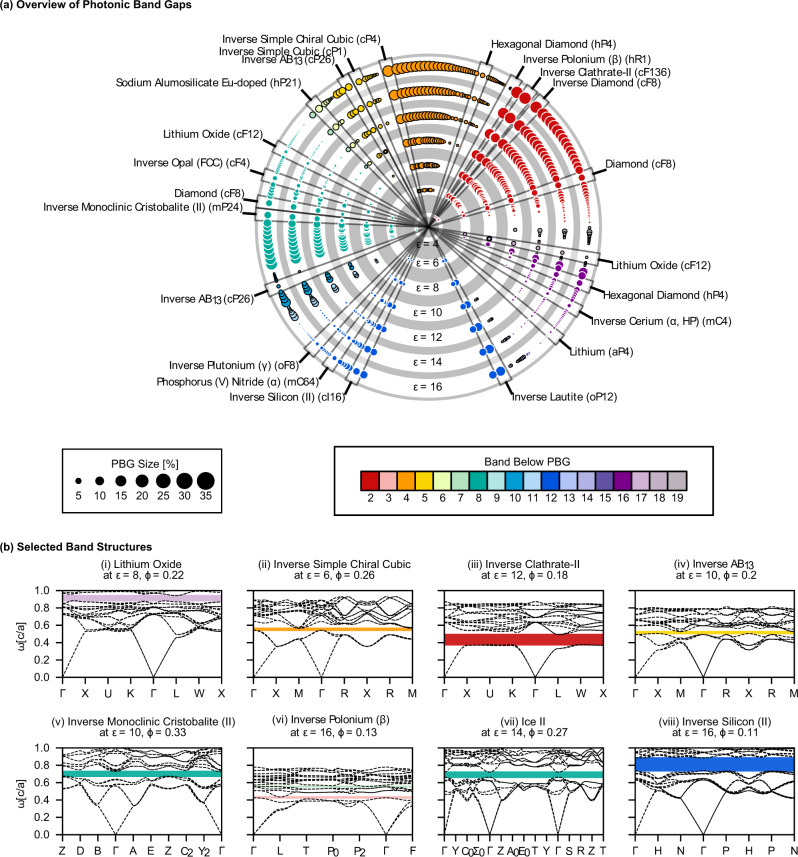
Overview of PBG properties of nature-inspired structural templates. **a** The largest PBGs in each PBG location found for each structure computed for *ε* = 4–16, arranged in concentric rings by *ε*. Circle areas are proportional to the PBG size; colors correspond to the band location of the PBG. Circles are arranged by structure, where each radius corresponds to a single structure at different *ε*. Some structures are shown more than once since some structures were found to exhibit PBGs in different locations at different filling fractions. Structures that have been previously studied or noted elsewhere in the text have been labeled. **b** Selected band structures, plotted for the first 20 bands across reciprocal space. (i) Lithium oxide, (ii) Inverse simple chiral cubic, (iii) Inverse clathrate II, (iv) Inverse *AB*_13_, (v) Monoclinic tridymite, (vi) Inverse *β*-polonium (the only template found to have a PBG between bands 3–4), (vii) Ice II, and (viii) Inverse silicon II.

Our data set consists of 2714 crystal structures from multiple sources^18,19^. Each structure is used as a template, with identical dielectric or air spheres on every lattice site for the "direct” or "inverse” versions, respectively. We screened each structure for PBGs between the first 20 bands across the structural parameters of volume filling fraction *ϕ* (from 0–1) and dielectric constant *ε* (from 4–16 in reduced units), resulting in 151,593 band structure calculations via MIT Photonic Bands (MPB)^1^ and managed with the *signac* data framework^20^. PBGs are reported as dimensionless percentages, i.e., the range of frequencies within the PBG (Δ*ω*) divided by the mid-gap frequency (ω^*^)^1^. Of the photonic band structures computed, 12,778 contain PBGs of size 0.1% or larger for 0.022 ≤ *ϕ* ≤ 0.711. The lowest *ε* resulting in a PBG is *ε* = 4 (agreeing with literature)^6,13^, and many structures have PBGs with values of *ε* as low as 6. In total, we find 474 unique PBGs >0.1% in 351 structures (some structures exhibit PBGs at more than one band location at different filling fractions). A summary of the PBGs is shown in Fig. 1, with the maximum gap size for each location (above band 2 and higher) indicated by the size and color of the circles. PBGs under the first band or between bands 1 and 2 are physically impossible, as the first two bands will approach zero frequency at the center of the BZ. The gap atlas, selected photonic band structures, and isosurface representations of structures mentioned in the main text are provided in the Supplementary Information. Photonic band structures for templates that are of special interest or mentioned later in the text are shown in Fig. 1b. Results were validated where possible against previous literature^1,6,10,12,21,22^. Validation data sets are provided in the Supplementary Information. The maximum PBG size (for any *ϕ* and with *ε* = 16) for the well-known diamond, inverse diamond, inverse opal, and inverse simple cubic are approximately 15, 34, 8, and 12% above bands 2, 2, 8, and 5, respectively.

**Fig. 2:**
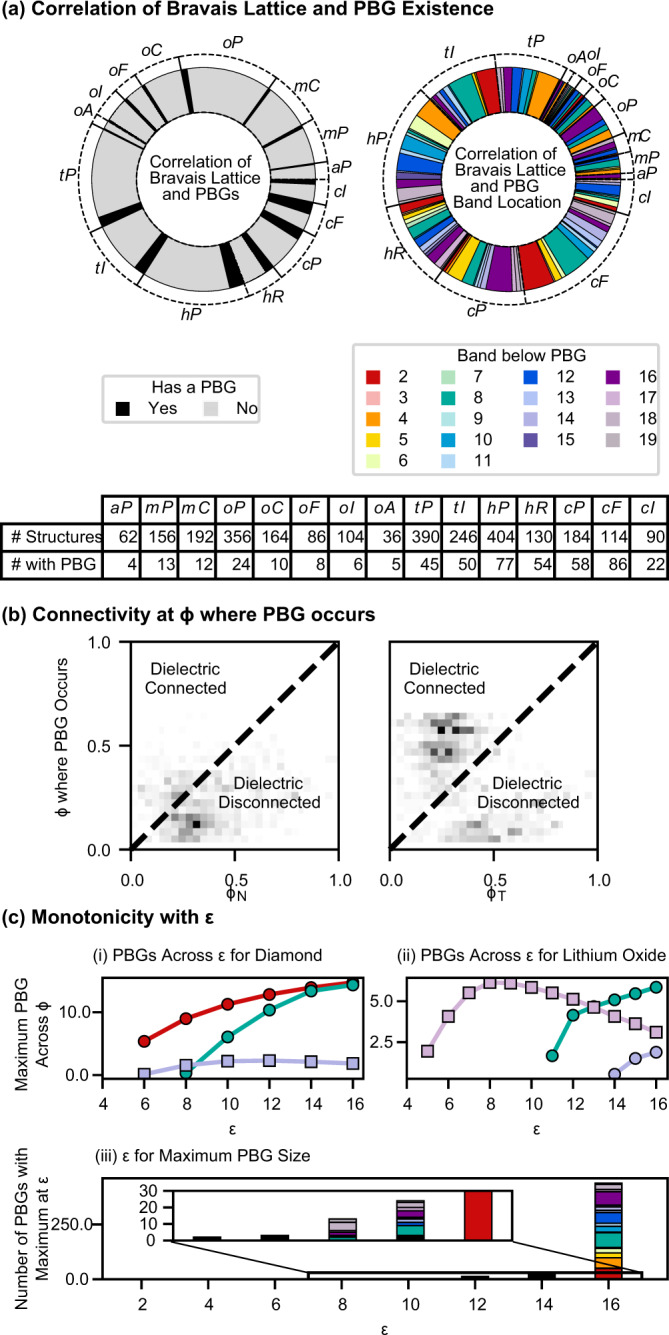
Correlation of PBG existence, size, and location with structural features. **a** Bravais Lattice (BL) having a PBG or a PBG above specific bands. A summary table for each BL is given. **b** Comparison of *ϕ* where PBGs occur with *ϕ*_*T*_ and *ϕ*_*N*_. Data above and below the dotted lines denote PBGs that occur when the dielectric is connected or disconnected, respectively. **c** Monotonicity with *ε*. (i and ii) Size of PBGS in diamond (i) and lithium oxide (ii) across *ε*. PBGs that are non-monotonic with *ε* are denoted with squares. (iii) *ε* where the maximum PBG size occurs, which is an inset showing the PBGs with maxima at low *ε*.

We find PBGs in all 14 Bravais lattices, including the more asymmetric monoclinic and triclinic lattices. In Fig. 2a, we find that PBGs most often exist in *cF* and *cI* lattices, concurring with the earlier postulate, as well as rhombohedral (*hR*) and cubic primitive (*cP*) lattices, despite their aspherical BZs. As expected, large PBGs (>25%) tend to occur in structures with diamond or gyroid-like topologies, but less expectedly, they also occur in *tI* lattices, which can have highly aspherical BZs (Fig. 2b(i)). We can compare the sphericity of the BZs using the isoperimetric quotient, defined as $\frac{36\pi {V}^{2}}{{A}^{3}}$, where IQ = 1 for perfect spheres. The IQ of *hR*, *cP*, and *tI* lattices are at most *π*/6 = 0.523, whereas the IQ of *cF* and *cI* lattices are 0.7533 and 0.7404, respectively. Thus a nearly spherical BZ is neither necessary nor sufficient to produce a PBG, although it will often correlate with the largest PBGs. Discussions of the point group symmetry of the structures and Wyckoff sites, sphericity of the BZ, space group, and angles between nearest-neighbor vectors are provided in the Supplementary Information.

The space group of the crystal strongly constrains the presence and locations of band crossings. The relationship between the crystal space group and mandatory band crossings is well understood^23^. However, the relationship between the space group and the absence of band crossings is nontrivial and of interest for band gap design^24^. Our dataset shows strong correlations between the Bravais lattice and gap locations. We find that a large fraction of PBGs between bands 2 and 3 are in *cF* and *tI* structures, and PBGs between bands 5 and 6 are primarily found in *cP* structures, as shown in Fig. 2a.

### Connectivity

Next, we look at the 2D heuristic of connectivity of the dielectric^7^. For any structure realized with monoatomic spheres on every lattice site, there are two connectivity thresholds defined by the geometry. The first, which we denote *ϕ*_*N*_, is min(*ϕ*) such that the dielectric forms a continuous network, defined for both direct and inverse structures. The second, which we denote *ϕ*_*T*_, is min(*ϕ*) such that the dielectric spheres on any two lattice sites touch and can only be defined for direct structures. A schematic of these thresholds is provided in the Supplementary Information. For all structures, *ϕ*_*T*_ ≤ *ϕ*_*N*_, and for some direct structures, *ϕ*_*N*_ = *ϕ*_*T*_ as the lattice sites are evenly distributed. As shown in Fig. 2b, we find that many PBGs, including large PBGs, occur for *ϕ* < *ϕ*_*N*_ and *ϕ* < *ϕ*_*T*_, especially in PBGs at high frequencies, as these modes can more easily travel between disconnected regions of the dielectric.

### Variation with dielectric strength (Epsilon)

Finally, in our dataset, we find 41 of 474 cases where the PBG size is non-monotonic with *ε*, as seen in Fig. 2d(iii). The most dramatic non-monotonicity occurs for lithium oxide (also known as the fluorite or the C1 structure), whose PBG between bands 17 and 18 increases for 6 ≤ *ε* ≤ 9 but decreases to half its maximum size for 9 ≤ *ε* ≤ 16, as seen in Fig. 2d(ii). The other PBG, between bands 8–9, was previously theorized in Maldovan et al.^11^ and is monotonic with *ε*. This non-monotonic relationship is potentially due to various factors, e.g., the location of the bandgap; whether the dielectric spheres are commensurate with the frequency of the PBG; occurring within a density regime where Mie scattering may increase the complexity of the mode configurations^25,26^. Also possible is that as the dielectric constant changes, the optimal topology changes, as suggested in Men, et al.^13^, and only for intermediate *ε* is an optimized geometry accessible. Similar non-monotonicity was observed in an experimental system and mentioned but not thoroughly investigated^27^. We note that all structures studied in this work are studied as perfect crystals, not results from self-assembly.

### Field analysis

In simple terms, a photonic band gap will occur when two neighboring electromagnetic modes exhibit modes with sufficiently different frequencies, which is proportional to their energy. The ways to engineer this difference in frequency are highly constrained with only 2D geometries. In 3D, the picture is understandably more complex. Here we parallel the field analysis done for 2D by Meade, et al.^7^ to understand the 3D analogs to the “dielectric” and “air” bands.

**Fig. 3:**
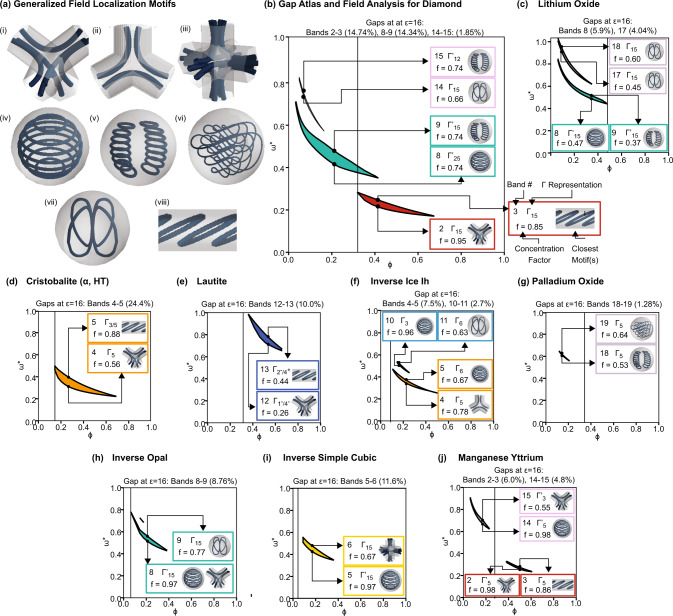
Mode configurations and motifs for PBG photonic crystals. **a** Common motifs in the mode conformations of PBG photonic crystals. Motifs (i–iii) exhibit regions of high electric energy density between adjacent unit cells, while the electric energy density for modes (iv–viii) is confined to an isolated region of dielectric material. **b** Gap atlas and field analysis of diamond, which has two large PBGs at different regions of filling fraction, either between bands 2–3 or 8–9, and a small PBG between bands 14–15. The dotted line denotes the filling fraction at which the dielectric has formed a continuous network. **c** Gap atlas and field analysis of lithium oxide, which has two PBGs at different regions of filling fraction, either between bands 8–9 or 17–18. The dotted line denotes the filling fraction at which the dielectric has formed a continuous network. **d**–**j** Gap atlases and field analyses for *α*-cristobalite (*cF*24), lautite (*oP*12), Ice Ih (*hP*30), palladium oxide (*tP*4), inverse opal (*cF*4), inverse simple cubic (*cP*1), and manganese yttrium (*tI*8).

We calculated the electric displacement field **D** above and below the gap for select structures found to have a PBG. From these vector fields, we simplified them into flow diagrams of the electric energy localization using the plotting package *Mayavi*.^28^ Several motifs (formally defined as general patterns or distinctive features) emerged from these flow diagrams, as shown in Fig. 3a(i–viii). More details on the reduction from vector fields to flow diagrams are provided in the Supplementary Information. We quantify the fraction of electric energy found in the dielectric with the concentration factor *f*, given by:

2 $$f=\frac{{\int}_{{V}_{\varepsilon }}{{\bf{E}}}^{* }({\bf{r}})\cdot {\bf{D}}({\bf{r}})\,dr}{\int {{\bf{E}}}^{* }({\bf{r}})\cdot {\bf{D}}({\bf{r}})\,dr},$$

where *V*_*ε*_ is the high dielectric region. For each PBG, we highlight four properties of the eigenmodes delineating the PBG: (1) band number, (2) Γ representation of the bands, (3) *f*, and (4) the motif that most closely resembles the displacement field lines. These properties are computed for the filling fraction that maximizes the PBG of a given structure and for the wavevectors **k** corresponding to the maxima and minima of the bands below and above the PBG, respectively. The dotted line signifies *ϕ*_*N*_ (for the structures shown, *ϕ*_*N*_ = *ϕ*_*T*_).

In 2D, the dielectric band typically has 50% or more of the electric energy density localized in the dielectric compared to the air band^7^. In contrast, from band 2 to 3 in diamond (Fig. 3b), the concentration factor *f* decreases by 0.1, i.e., 10% more of the electric energy is in the dielectric in band 2 than band 3. There is no change in concentration factor between bands 8 and 9, and the concentration factor of band 15 is higher than that of band 14. Thus, band 15 is more of a “dielectric band” than band 14.

The motifs of electric energy localization in 3D delineating a band gap do not fall neatly into the categories of “dielectric” and “air” bands. Instead, they fall into two broad categories: those that travel between adjacent unit cells (Fig. 3a(i–iii)) and those that remain confined to a single region of dielectric (Fig. 3a(iv–viii)). For modes with field conformations similar to Fig. 3a(i–iii), increasing the connectivity of the dielectric reduces the mode frequency. Therefore if this motif is found in the band below a gap (but not above the gap), increasing connectivity will enlarge the PBG, as seen in the PBG between bands 2–3 in Fig. 3b. However, if both modes or the mode above the gap exhibit this type of motif, increasing the connectivity will reduce or close the PBG, such as the 8–9 and 14–15 PBG in Fig. 3b.

We can use a similar analysis to explain the relationship between *ε* and PBG size in the lithium oxide crystal structure shown in Fig. 2b. The heuristic of monotonicity of gap size with *ε* assumes a mode configuration where the PBG is enlarged by higher localization in the dielectric, e.g., when there is a distinct air band and dielectric band. However, as the PBG in lithium oxide decreases as the filling fraction approaches the connectivity threshold, this assumption is invalid, and the relationship of Δ*ω*/ω^*^ and *ε* is complex.

## Discussion

Our data-driven exploration of the possible space of photonic band gap crystal structures shows that for many photonic crystals, it is clear that no single design rule applies to all PBGs. Many current heuristics provide general guidance to designing crystals for photonic band gaps, but we find many interesting counter-examples for every heuristic. Consequently, further inquiry is needed to predict PBGs in 3D, especially at higher frequencies, where these design rules most typically fail.

The 291 new PBG structures reported here should make for interesting targets, in particular for colloidal systems where diamond and similar structures have been difficult to synthesize, only recently realized with tetrahedral clusters of patchy particles^29^. Several of these structures shown to have PBGs are known to assemble on colloidal length scales: lithium oxide (6% in direct form) and simple chiral cubic (3.4% PBG in direct form, 27% PBG in inverse form) can be self-assembled using polyhedral nanoparticles^30,31^, the Clathrate II structure (33% PBG in inverse form) was recently found using DNA-programmed self-assembly^32^, and the *AB*_13_ structure (13.3% PBG in inverse form) is easily achievable with binary mixtures of hard spheres^33,34^. Furthermore, there are structures, previously unstudied in the colloidal community, that can be new targets for colloidal self-assembly, such as *tI*8^35^ (6.0% PBG in direct form, 25% PBG in inverse form) or *cI*16^36^ (6.4% PBG in direct form, 18.4% PBG in inverse form, the latter of which was previously reported^37,38^). However, the choice of structure is largely fabrication- and application-dependent and is often best decided by those trying to realize the structure. We have developed an open-source database of photonic structures, which can be used to browse and download the structures detailed in the study so that further researchers can gain inspiration and insight.

## Methods

### Data management

The data for this project was managed using signac, and the workflow was managed by signac-flow^20^ in a multi-level project. The top level of the project consists of statepoints associated with the structural data. Inside each structure statepoint was an additional project managing the statepoints containing radii and dielectric constant.

### Structure retrieval and conversion

Crystallographic Information Files (".cif” format) were downloaded from the Crystallographic Open Database (COD) and the Inorganic Crystallographic Structure Database (ICSD)^18,19^. Information regarding atomic constituents and structure name was taken directly from the *.cif* files, regardless of the correctness or conditions of the original authors’ publication or data. Additionally, we included structures found in unrelated simulations in the Glotzer group, some of which have no natural analog. These position files were then converted to a set of lattice vectors and a fractional basis, with the first lattice vector normalized to unit length.

### Input parameters for MPB

All PBG calculations were conducted using MPB, a scheme-based eigenmode solver developed at MIT^1^. MPB calculates the photonic band structure through iterative planewave eigenmode searching. In MPB, the input parameters required are: (1) lattice vectors and fractional basis, (2) particle radius and dielectric constant, (3) fractional *k*-point path in reciprocal space, (4) *k*-point interpolation, (5) resolution, (6) mesh size, and (7) number of bands to calculate.

(1) and (2) define the statepoints for this study. (3) was generated using the procedure below. (4) was set to 16 in the initial screening, and set to 32 in complex structures or during the second round of screening. (5) was set to 1 in the initial screening, and 6 during the second round of screening. (6) was set to 5. (7) was set to 20. Only those statepoints with PBGs during initial screening were included in the second round of screening. Those structures found not to have a PBG with minimal k-points would not have a PBG with additional k-points, which would only reduce or close the calculated PBG.

### Radius screening

For each structure, particle radii were initialized to *r* ∈ (0.0, 1.5) and Δ*r* = 0.01 and calculations run. MPB outputs were then queried, and radii with filling fractions outside of [0.0, 1.0] for a structure were removed. For each structure, radii were added uniformly at smaller intervals where necessary, such that each structure was screened with at least 20 radii for both dielectric spheres and air spheres.

### Dielectric constants

For each structure, we initially assumed a dielectric constant *ε* = 16, the highest theorized dielectric constant for solid, translucent media^39^. It has been postulated that any crystal will exhibit a PBG in the limit of infinite dielectric constant^8^, thus we tested a wide variety of crystals, even if difficult to realize in experiment. After the initial computations at *ε* = 16, we performed computations for structures and statepoints found to have PBGs at lower *ε*, down to *ε* = 2 or such that Δ*ω* = 0.

### k-point path generation

The first Brillouin zone was computed by finding the Voronoi tessellation of the reciprocal lattice for each structure using the Voronoi functionality of SciPy^40^. The path through the BZ was taken to be a highly redundant traversal along the edges of each face of the surface of the BZ, including to and from the face center and the Γ-point. This path, though redundant, is guaranteed to exhaustively sample the high symmetry points at which band extrema occur. An example of a highly redundant k-point path is provided in the Supplementary Information.

For field analysis, the irreducible BZ was calculated to ensure correct k-point labels in the calculation of Γ representation.

### Structural analysis

In order to calculate the connectivity thresholds and bond angles, we used the Python package pymatgen^41^, an open-source materials analysis package.

For every structure, we computed the radius *r* and filling fraction *ϕ* for up to two different thresholds, (1) corresponding to the first peak in the radial distribution function (RDF), denoted *ϕ*_*T*_, and (2) corresponding to the radius at which the spheres on the lattice sites are connected in a continuous network, denoted *ϕ*_*N*_. *ϕ*_*T*_ is ill-defined for inverse structures. (2) was found for direct structures using pymatgen and by analyzing the voxelizations of inverse structures using the *skimage* package^40^. For some direct structures, these thresholds are the same.

Space groups and symmetry information were found using the open-source package spglib^42^ and 3D visualization was done with the open-source package *mayavi*^28^.

### Field analysis

For MPB, there is an option to output field files for a given computation at every $\overrightarrow{k}$ and band. We took the electric fields in these files and visualized them using *mayavi*^28^. The flow reduction was made using 1–2 unit cells of the structure with a resolution of 24–40 grid points. Motifs were drawn based upon analysis of these flow diagrams. A schematic of this process is provided in the Supplementary Information.

The concentration factor was computed using (2) as described in Meade, et al.^7^.

Γ classification was done using character tables found in the Bilbao Crystal Database^43^ and Dresselhaus, et al.^23^.

## Supplementary information

Supplementary Information

Description of Additional Supplementary Files

Supplementary Data 1

## Data Availability

Data for all PBG structures and MPB input parameters is publicly available at https://glotzerlab.engin.umich.edu/photonics/. A summary of the structures analyzed in this study is provided in Supplementary Data 1. Further data generated during and/or analyzed during the current study are available from the corresponding author on reasonable request.
