# Supplementary material for: The diversity of three-dimensional photonic crystals
Source: Nat Commun. 2021 May 5;12:2543. doi: 10.1038/s41467-021-22809-6 (PMC8100142; doi:10.1038/s41467-021-22809-6)
Supplement: Supplementary file 1 — Supplementary Information [file 41467_2021_22809_MOESM1_ESM.pdf]

Unexpected Diversity of Three-Dimensional Photonic Crystals: Supplementary Information

Rose K. Cersonsky,[1] James Antonaglia,[2] Bradley D. Dice,[2] and Sharon C. Glotzer[1,2,3,4,5]

[1] *Macromolecular Science and Engineering Program, University of Michigan, Ann Arbor, MI 48109*

[2] *Department of Physics, University of Michigan, Ann Arbor, MI 48109*

[3] *Department of Chemical Engineering, University of Michigan, Ann Arbor, MI 48109*

[4] *Department of Materials Science and Engineering, University of Michigan, Ann Arbor, MI 48109*

[5] *Biointerfaces Institute, University of Michigan, Ann Arbor, MI 48109*

(Dated: 26 March 2021)

CONTENTS

I. Supplementary Note 1 4

A. Summaries of Data 4

B. Method Figures 5

1. Comparison with Previously Published Data 5
2. Data Management with Signac 7

C. Extended Analysis 9

1. Bravais Lattice and Gap Location 9
2. Correlation with PBG Existence 10
3. Correlation with Large PBGs 11
4. Relative Probabilities Across Space Groups 12
5. Effects of ϕ and ε 13

II. Supplementary Note 2 14

A. *aP*4-Li (Inverse) 14

B. *cF*12-Li_2O (Direct) 15

C. *cF*136-Si (Inverse) 17

D. *cF*24-SiO_2 (Direct) 18

E. *cF*4-Cu (Inverse) 19

F. *cF*8-C (Direct) 20

G. *cF*8-C (Inverse) 22

H. *cP*1-Po (Inverse) 24

I. *cP*24-SiO_2 (Direct) 25

J. *cP*4-X (Inverse) 26

K. *oP*12-CuAsS (Direct) 27

L. *tI*16-$CoSiCu_2S_4$ (Direct) 28

M. *tI*8-YMn_2 (Direct) 29

N. *tP*12-SiO_2 (Direct) 31

O. *tP*4-PdO (Direct) 32

P. *hP*21-$NaAlSiO_4$ (Direct) 34

Q. *cP*26-UBe_{13} (Inverse) 35

R. *mP*24-SiO_2 (Inverse) 37
S. *oF*8-Pu (Inverse) 38
T. *mC*64-P_3N_5 (Direct) 39
U. *cI*16-Si (Inverse) 40
V. *mC*4-Ce (Inverse) 41
W. *hP*4-Ge (Direct) 42
X. *hR*1-Po (Inverse) 44
Y. *oC*48-H_2O (Direct) 46
Z. *hP*20-H_3O (Inverse) 47

Supplementary References 48

I. SUPPLEMENTARY NOTE 1

A. Summaries of Data

Another way of viewing Fig. 1 from the main text is to organize in terms of PBG size and ε, as shown in **1**.

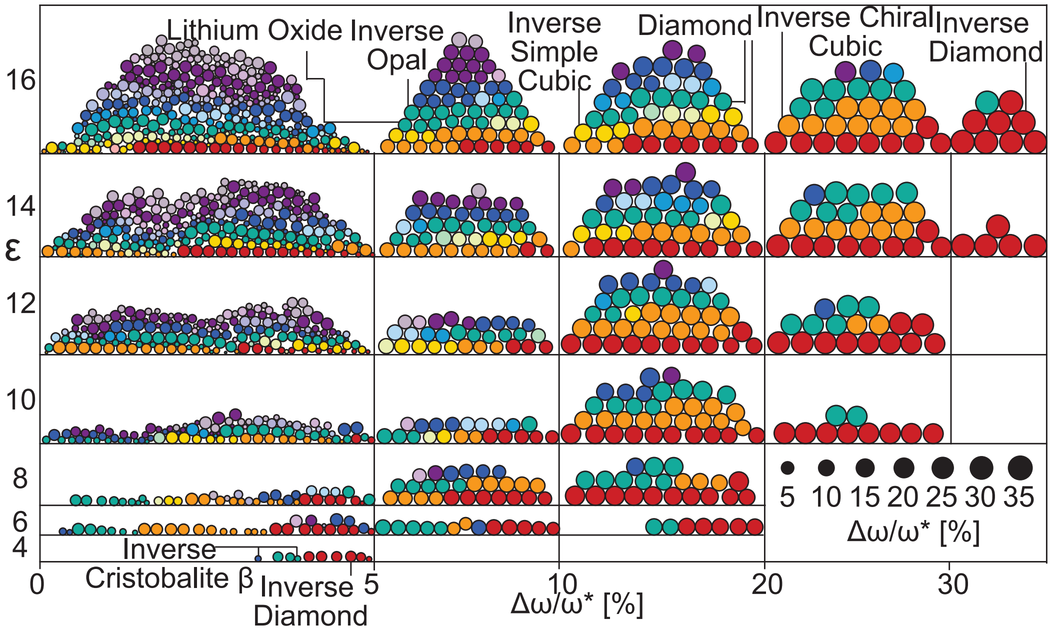

Band Below PBG	2	3	4	5	6	7	8	9	10	11	12	13	14	15	16	17	18	19
Color																		
# PBGs (ε=16)	48	1	47	24	14	4	74	3	25	13	48	13	19	8	64	14	25	6

Supplementary Figure 1. **PBGs Properties of Nature-Inspired Structural Templates**. Largest PBGs found for each structure generated among $\varepsilon = 4$–16. Circle areas are proportional to the PBG size; colors correspond to the location of the PBG. Some structures are shown more than once, as some structures were found to exhibit PBGs in two different locations at different filling fractions. Structures that have been previously studied or noted in the main text have been labeled.

B. Method Figures

1. *Comparison with Previously Published Data*

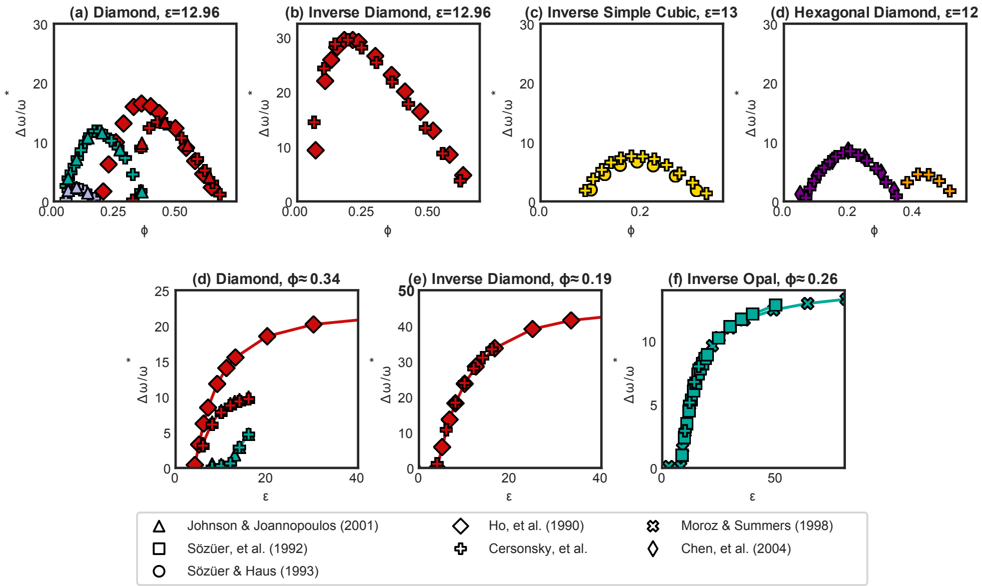

Supplementary Figure 2. Comparison of Reported and Previously Published Data for Fixed ε and ϕ. Color indicates bands between which the PBG occurs, with plotting marker corresponding to data source. (a) PBG between bands 2-3 (red), 8-9 (teal) and 14-15 (lavender) for Diamond at $\varepsilon = 12.96$, as reported by[1] and generated by the example code given by[4]. PBG sizes reported in Ref.[1] were found to be overestimated in[5], therefore a better benchmark for diamond is provided by[4]. (b) PBG between bands 2 and 3 in Inverse Diamond at $\varepsilon = 12.96$, as reported by[1]. (c) PBG between bands 5 and 6 in Inverse Simple Cubic at $\varepsilon = 13$, as reported by[2]. (d) PBG between bands 16 and 17 in Hexagonal Diamond at $\varepsilon = 12$, as reported by[3]. PBG between bands 4 and 5 (orange) were unreported in[3], with no indication if calculations for corresponding ϕ were run. (e) PBG between bands 2-3 (red) and 8-9 (teal) for Diamond at $\phi \approx 0.34$, as reported by[1] and generated by the example code given by[4]. PBG sizes reported in Ref.[1] were found to be overestimated in[5], therefore a better benchmark for diamond is provided by[4]. (f) PBG between bands 2 and 3 in Inverse Diamond at $\phi \approx 0.19$, as reported by[1]. (g) PBG between bands 8 and 9 in Inverse Opal at $\phi \approx 0.26$, as reported by[5,6].

In Fig. **2**, we compare the PBG sizes computed using our methodology and previously published in literature within references[1–6]. Any discrepancies between the computations reported and previous literature can be due to differing smoothing functions of ε space, using a more exhaustive IBZ, and small errors in transcribing previous literature, which were available in figure, and not

table, form.

2. *Data Management with Signac*

The data for this project was managed using signac and the workflow managed by signac-flow in a multi-level project.[7] The top level of the project consisted of statepoints consisting of structural data. Inside each structure statepoint was an additional project managing the statepoints containing radii and dielectric constant.

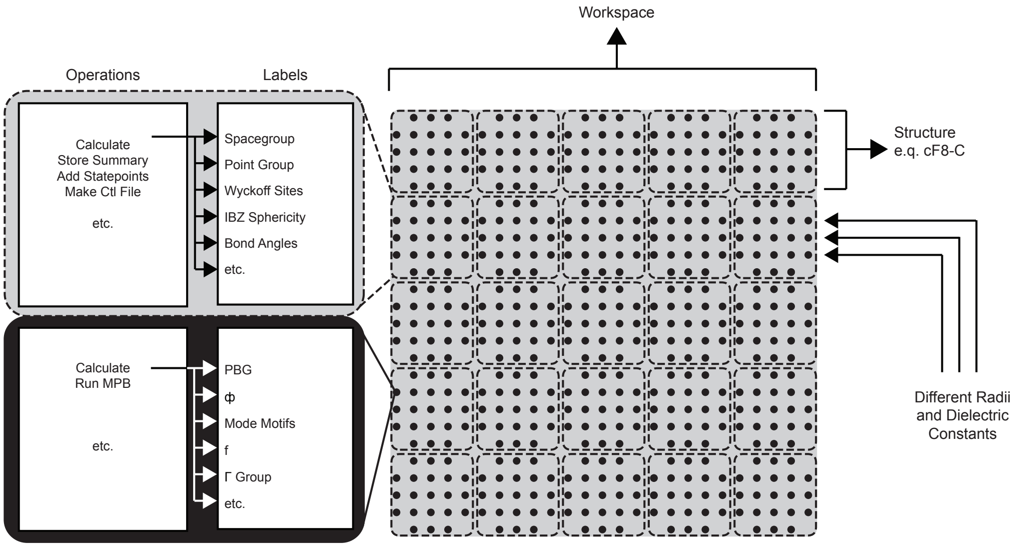

Supplementary Figure 3. **Organization of Project Using *signac* and *signac-flow***. Operations and labels were stored individually for the structure at large and independent radii and dielectric constants, as was appropriate. Typical structure-level operations included symmetry calculations, PBG atlas generation, and summarizing lower level data. Typical lower level operations include running and analyzing MPB, computing fill fraction, and field analysis.

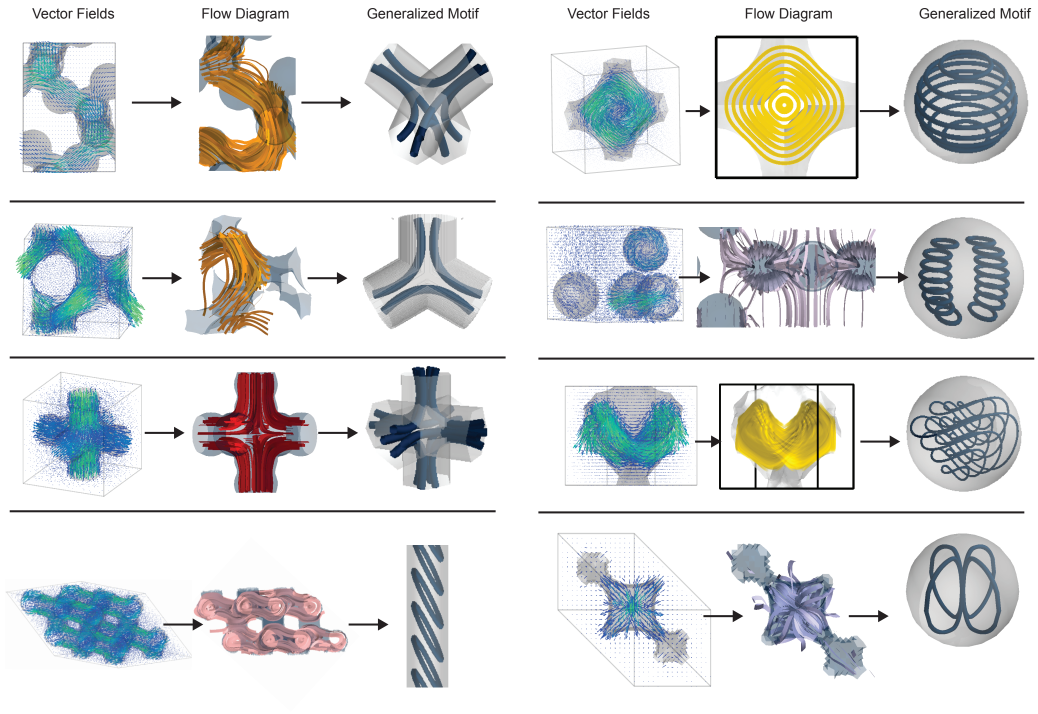

Supplementary Figure 4. **Schematic of Complexity Reduction from Vector Fields to Mode Motifs**. For each mode motif in the main text, we show the reduction in visual complexity from the vector fields to a flow diagram to a mode motif. For each motif we attempted to provide a visual that best represents the motif, with many structures exhibiting variations on these motifs or mixed motif behavior.

C. Extended Analysis

For this section, we will report percentages of structures that have a particular structural fingerprint across all structures studied and the 351 that have PBGs. We will also report relative probabilities, *i.e.* the probability a given fingerprint is found in structures with PBGs divided by its probability of being found in all structures. We compared the sample of structures used in this project with a rough estimate of the data space available through ICSD and COD to show that our sample set can represent a general distribution.

1. Bravais Lattice and Gap Location

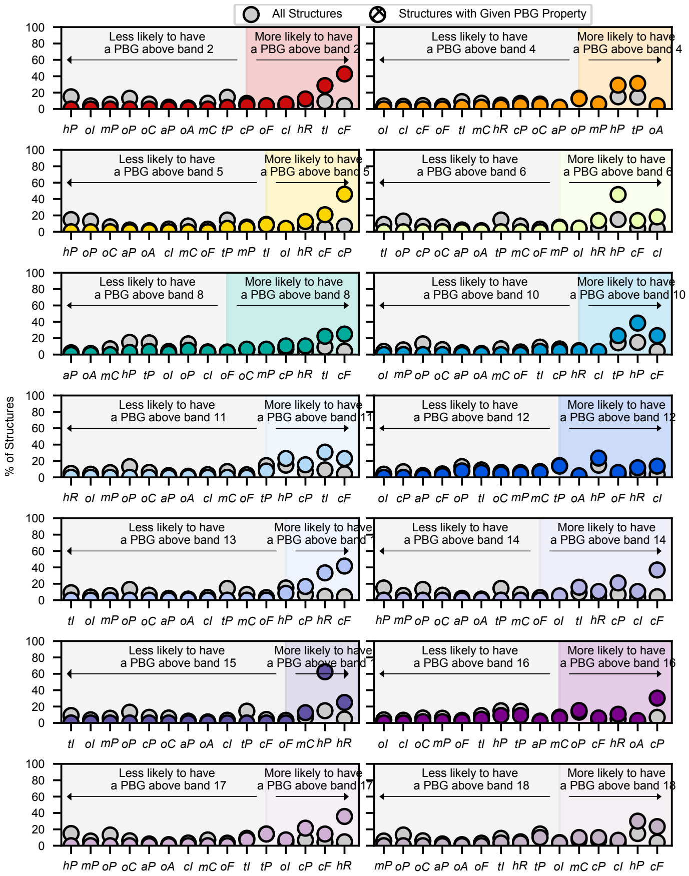

Supplementary Figure 5. **Correlation between PBG Location and Bravais Lattice**. Here we show an extension of Fig. 2(a)(iii-iv) from the main text, including PBG locations where there was less clear correlation between Bravais Lattice and PBG location.

2. Correlation with PBG Existence

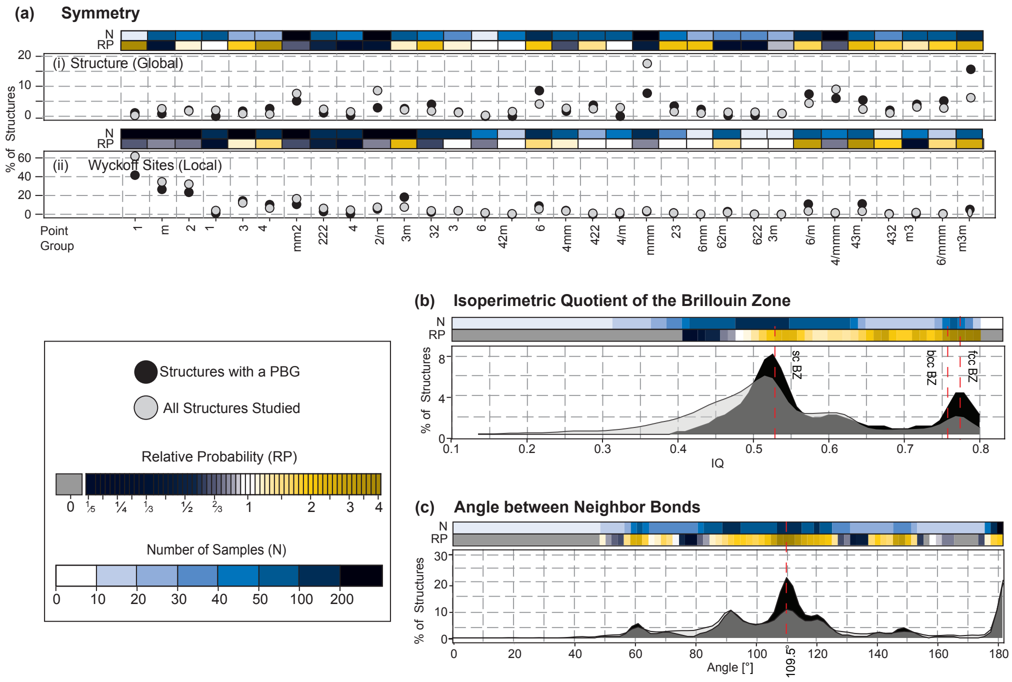

Supplementary Figure 6. **Comparison of Structural Features for PBG Structures and All Structures**. For each subfigure, the grey and black markers/area denote values for all structures and those with PBGs, respectively. (a) Point group symmetry of the (i) structure and (ii) Wyckoff sites, increasing in symmetry order to the right. (b) Isoperimetric quotient of the first Brillouin zone (BZ). Isoperimetric quotient is a measure of sphericity, close to 0 for highly non-spherical shapes and 1 for spheres. The values for cubic structures, which are constant for all structures of a given centering, are denoted with red dotted lines. (c) Measure of the angle between neighboring "bonding" sites. For each lattice site, we computed this value by considering the first shell of nearest neighbor sites.

3. Correlation with Large PBGs

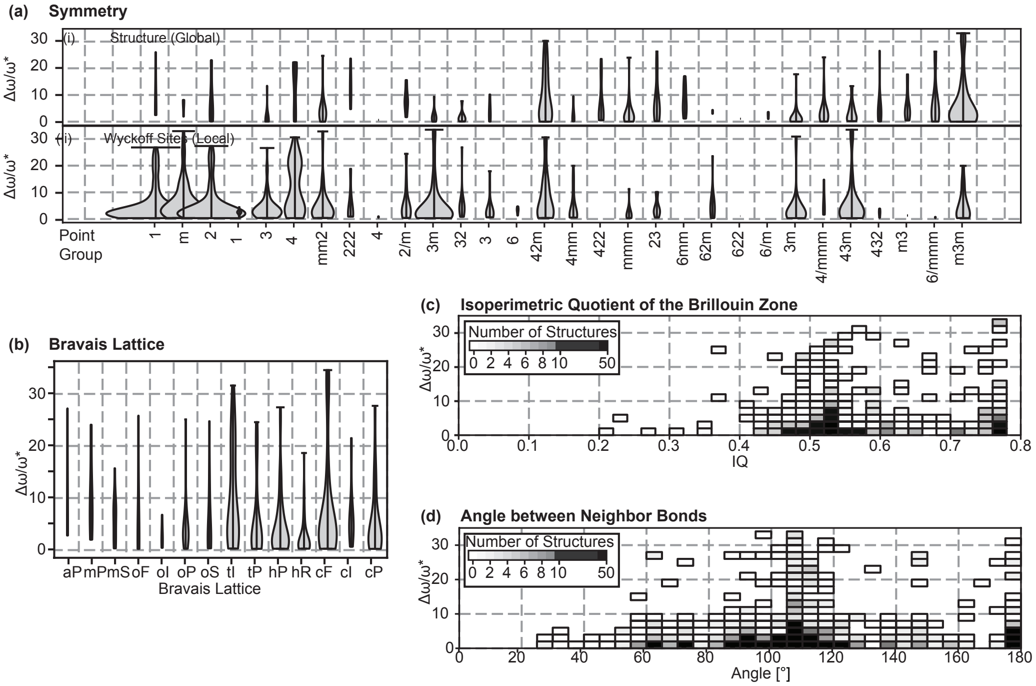

Supplementary Figure 7. **The Correlation of Structural Features with Large PBGs**. (a) The distribution of PBGs with respect to lattice setting and PBG size, with the Bravais lattices increasing in symmetry order to the right. (a) Distribution of PBGs with respect to (i) point group of the structure or (ii) point group of individual Wyckoff sites and PBG size, with the point groups increasing in symmetry order to the right. (b) Heat map of sphericity of the BZ and PBG size. (c) Heat map of bond angles and PBG size.

4. Relative Probabilities Across Space Groups

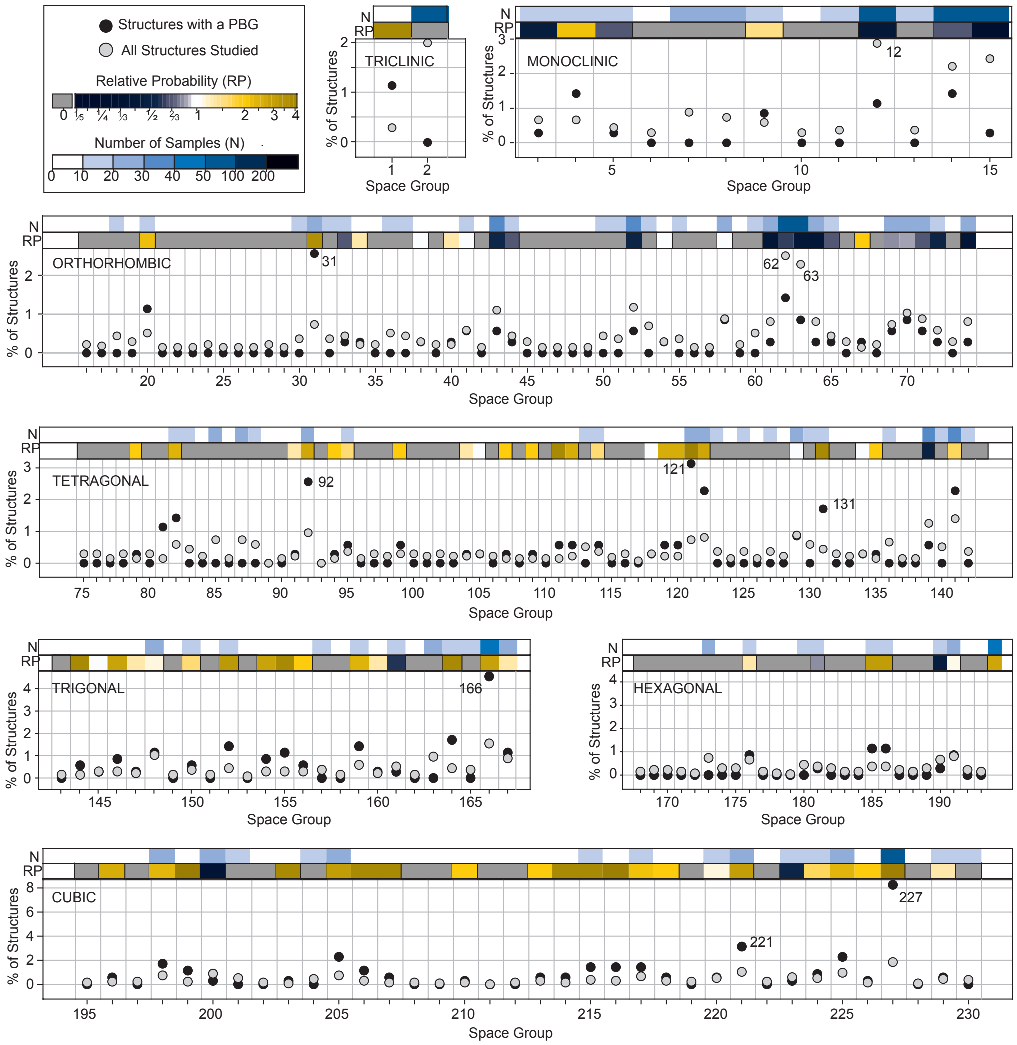

Supplementary Figure 8. **Relative Probabilities for Space Group Numbers**. Of the 230 total space groups, structures were calculated in the available 227 space groups. Space groups are separated by lattice group, with blue and yellow colormaps denoting the sample size and relative probabilities, respectively. Grey and black circles correspond to percentages of structures across the entire dataset and those with PBGs, respectively. Space group numbers with high or low relative probability are individually labelled.

5. *Effects of ϕ and ε*

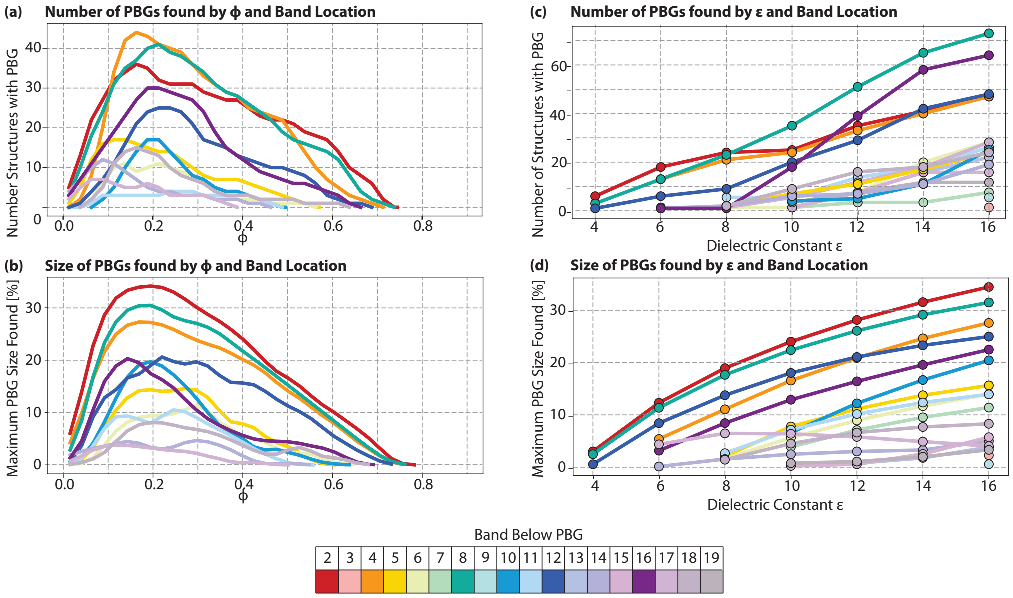

Supplementary Figure 9. **Summary of PBGs across Different Filling Fractions and Dielectric Constants**. (a) Number of PBGs found across filling fractions from 0 to 1. (b) Largest PBGs found at each filling fraction. (c) Number of PBGs found across dielectric constants, from 4–16. (d) Largest PBGs found at each dielectric constant. Each plot is separated by band number indicated by the color guide.

II. SUPPLEMENTARY NOTE 2

A. *aP*4-Li (Inverse)

Inverse Lithium

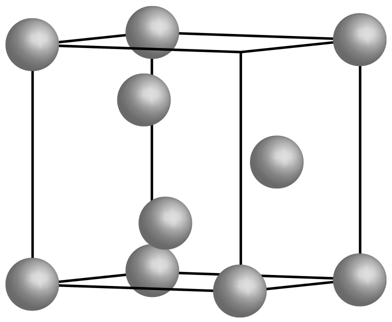

Image of *aP*4-Li, generated by Vesta

$\boldsymbol{a_1} = \ \hat{x} + 0.0001\ \hat{y} - 0.0004\ \hat{z}$
$\boldsymbol{a_2} = 1.0009\ \hat{y}$
$\boldsymbol{a_3} = 1.0003\ \hat{z}$

Space Group: 1 **Point Group:** 1

Found in Simulation

Structure DOI: 10.1021/nn204012y

Photonics DOI: 10.1063/1.1635664

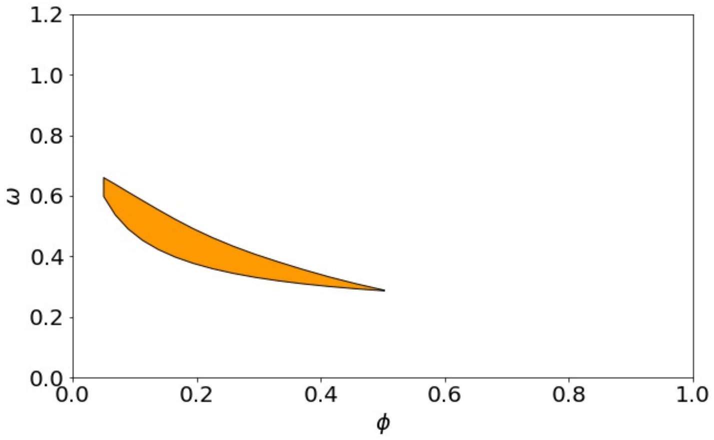

Supplementary Figure 10. Gap Atlas across filling fraction ϕ and frequency ω

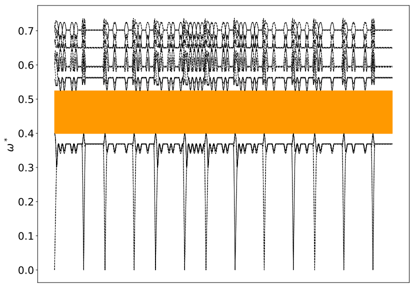

Band Structure across 1st BZ

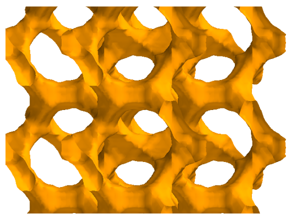

View along a_1

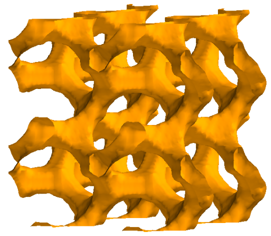

View rotated 140° about l_3 and elevated 0∘

Supplementary Figure 11. Band Structure and Isosurface of *aP*4-Li (Inverse) at radius = 0.4, filling fraction = 0.164, where the largest gap between bands 4 and 5 occurs with gap size 27.07%.

B. $cF12$-Li_2O (Direct)

Lithium Amide

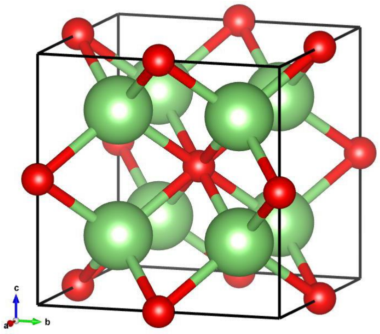

Image of $cF12$-Li_2O, generated by Vesta

$$\boldsymbol{a_1} = 1/\sqrt{2}\,\hat{y} + 1/\sqrt{2}\,\hat{z}$$
$$\boldsymbol{a_2} = 1/\sqrt{2}\,\hat{x} + 1/\sqrt{2}\,\hat{z}$$
$$\boldsymbol{a_3} = 1/\sqrt{2}\,\hat{x} + 1/\sqrt{2}\,\hat{y}$$

Space Group: 225 **Point Group:** $m\bar{3}m$

Crystallographic Open Database #4121514

Structure DOI: 10.1021/ja066016s

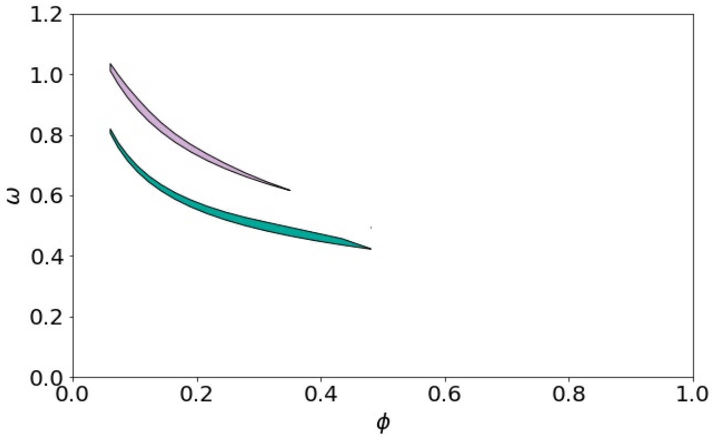

Supplementary Figure 12. Gap Atlas across filling fraction ϕ and frequency ω

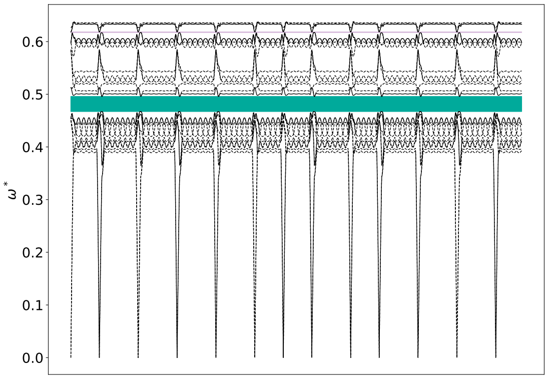

Band Structure across 1st BZ

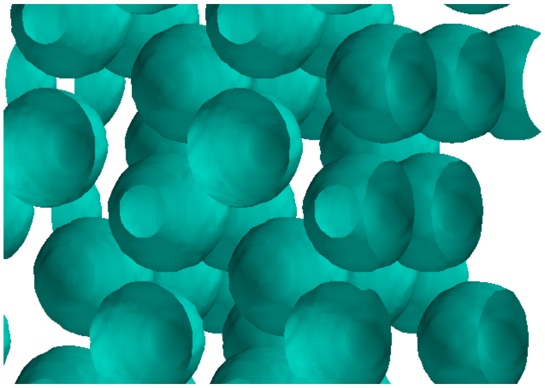

View along a_1

Supplementary Figure 13. Band Structure and Isosurface of $cF12$-Li_2O (Direct) at radius = 0.27, filling fraction = 0.278, where the largest gap between bands 8 and 9 occurs with gap size 5.93%.

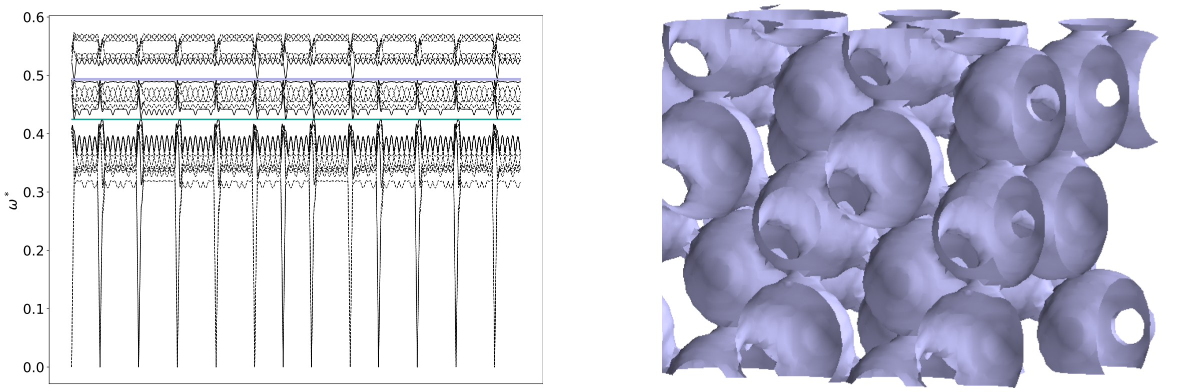

Band Structure across 1st BZ

View along a_1

Supplementary Figure 14. Band Structure and Isosurface of $cF12$-Li_2O (Direct) at radius = 0.3, filling fraction = 0.39, where the largest gap between bands 14 and 15 occurs with gap size 0.71%.

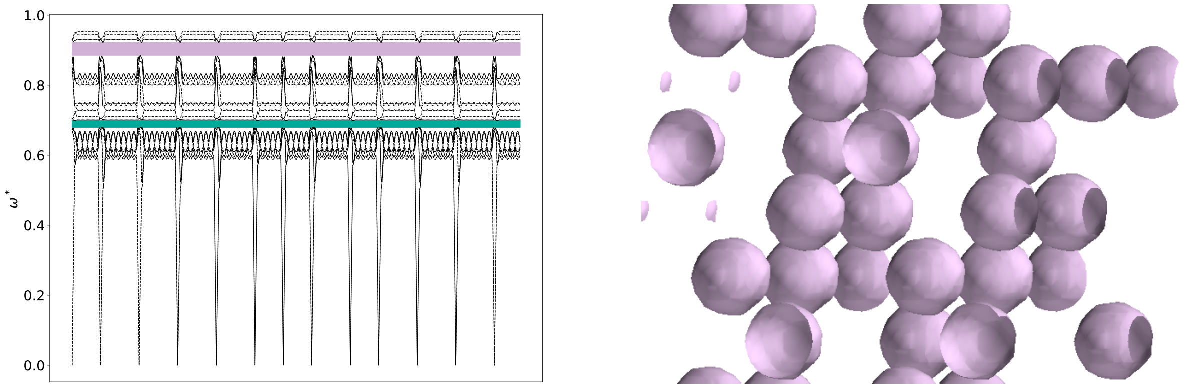

Band Structure across 1st BZ

View along a_1

Supplementary Figure 15. Band Structure and Isosurface of $cF12$-Li_2O (Direct) at radius = 0.18, filling fraction = 0.073, where the largest gap between bands 17 and 18 occurs with gap size 4.04%.

C. *cF*136-Si (Inverse)

Inverse Clathrate-II

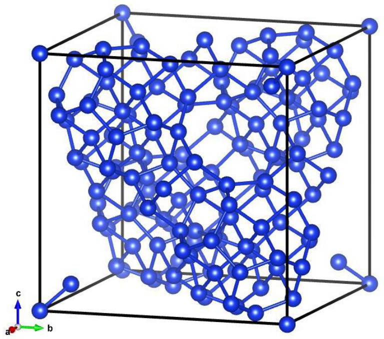

Image of *cF*136-Si, generated by Vesta

$$\boldsymbol{a_1} = 1/\sqrt{2}\,\hat{y} + 1/\sqrt{2}\,\hat{z}$$
$$\boldsymbol{a_2} = 1/\sqrt{2}\,\hat{x} + 1/\sqrt{2}\,\hat{z}$$
$$\boldsymbol{a_3} = 1/\sqrt{2}\,\hat{x} + 1/\sqrt{2}\,\hat{y}$$

Space Group: 227 **Point Group:** $m\bar{3}m$

Inorganic Crystallographic Database #56721

Structure DOI: 10.1103/PhysRevB.60.950

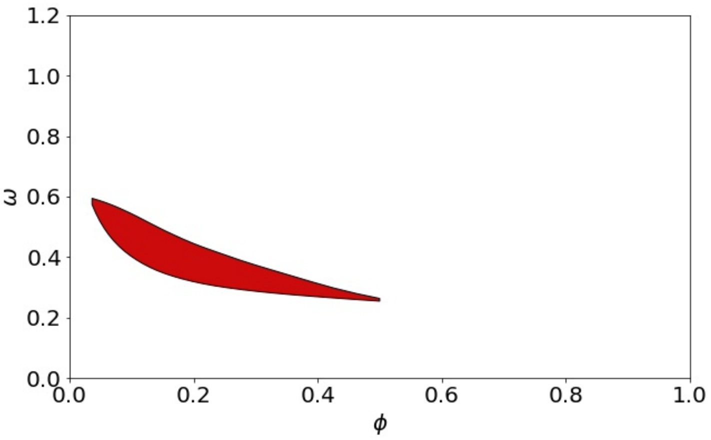

Supplementary Figure 16. Gap Atlas across filling fraction ϕ and frequency ω

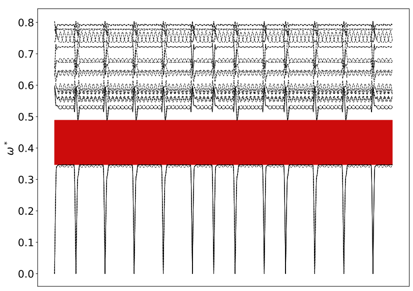

Band Structure across 1st BZ

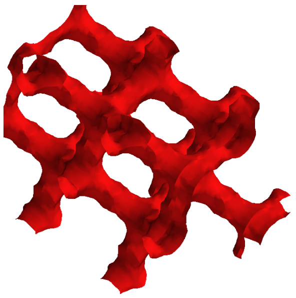

View along a_1

Supplementary Figure 17. Band Structure and Isosurface of *cF*136-Si (Inverse) at radius = 0.21, filling fraction = 0.153, where the largest gap between bands 2 and 3 occurs with gap size 33.9%.

D. $cF24$-SiO_2 (Direct)

Cristobalite (β)

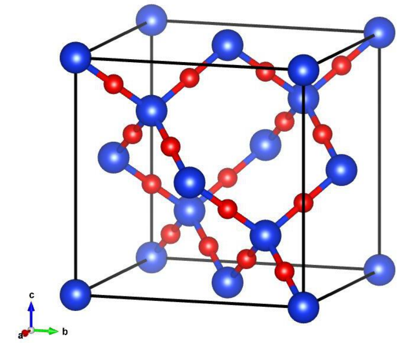

Image of $cF24$-SiO_2, generated by Vesta

$$a_1 = 1/\sqrt{2}\,\hat{y} + 1/\sqrt{2}\,\hat{z}$$
$$a_2 = 1/\sqrt{2}\,\hat{x} + 1/\sqrt{2}\,\hat{z}$$
$$a_3 = 1/\sqrt{2}\,\hat{x} + 1/\sqrt{2}\,\hat{y}$$

Space Group: 227 **Point Group:** $m\bar{3}m$

Inorganic Crystallographic Database #77460

Structure DOI: 10.1524/zkri.1992.201.1-2.125

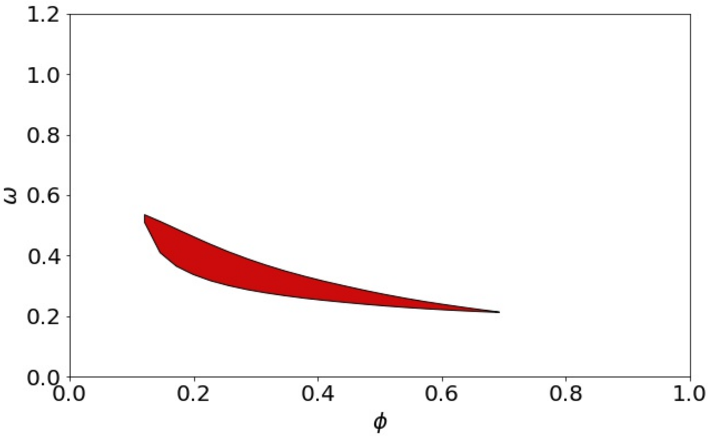

Supplementary Figure 18. Gap Atlas across filling fraction ϕ and frequency ω

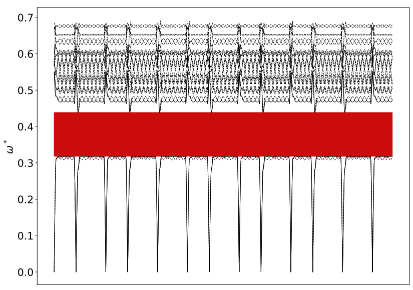

Band Structure across 1st BZ

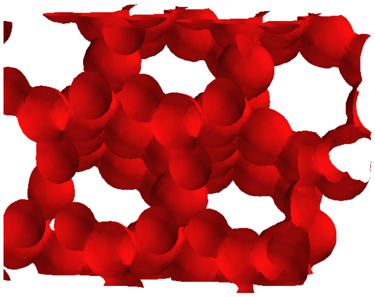

View along a_3

Supplementary Figure 19. Band Structure and Isosurface of $cF24$-SiO_2 (Direct) at radius = 0.19, filling fraction = 0.227, where the largest gap between bands 2 and 3 occurs with gap size 31.79%.

E. *cF*4-Cu (Inverse)

Inverse Opal (FCC)

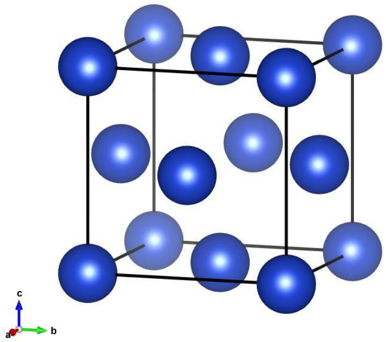

Image of *cF*4-Cu, generated by Vesta

$$\boldsymbol{a_1} = 1/\sqrt{2}\,\hat{y} + 1/\sqrt{2}\,\hat{z}$$
$$\boldsymbol{a_2} = 1/\sqrt{2}\,\hat{x} + 1/\sqrt{2}\,\hat{z}$$
$$\boldsymbol{a_3} = 1/\sqrt{2}\,\hat{x} + 1/\sqrt{2}\,\hat{y}$$

Space Group: 225 **Point Group:** $m\bar{3}m$

Inorganic Crystallographic Database #43493

Structure DOI: 10.1063/1.1728392

Photonics DOI: 10.1103/PhysRevLett.63.1950

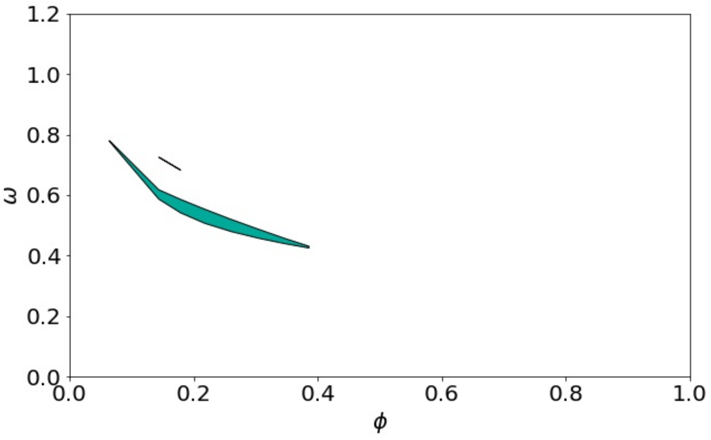

Supplementary Figure 20. Gap Atlas across filling fraction ϕ and frequency ω

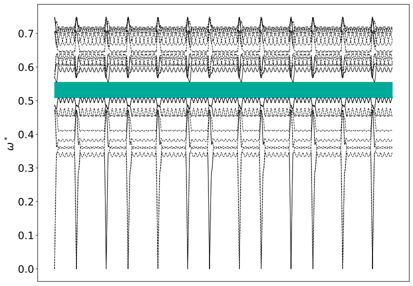

Band Structure across 1st BZ

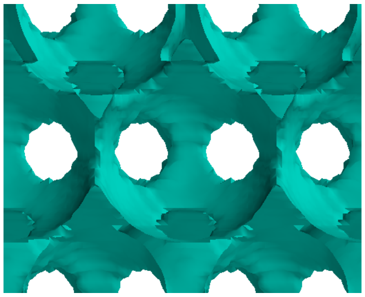

View along a_2

Supplementary Figure 21. Band Structure and Isosurface of *cF*4-Cu (Inverse) at radius = 0.51, filling fraction = 0.217, where the largest gap between bands 8 and 9 occurs with gap size 8.76%.

F. *cF*8-C (Direct)

Diamond

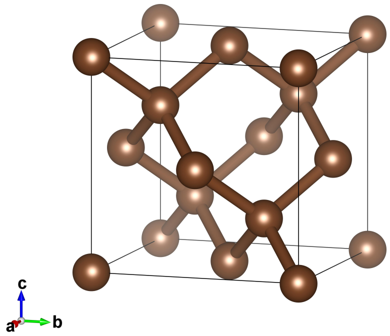

Image of cF8-C, generated by Vesta

$$a_1 = 1/\sqrt{2}\,\hat{y} + 1/\sqrt{2}\,\hat{z}$$
$$a_2 = 1/\sqrt{2}\,\hat{x} + 1/\sqrt{2}\,\hat{z}$$
$$a_3 = 1/\sqrt{2}\,\hat{x} + 1/\sqrt{2}\,\hat{y}$$

Space Group: 227 **Point Group:** $m\bar{3}m$

Inorganic Crystallographic Database #52054

Structure DOI: 10.1107/S0108768195010810

Photonics DOI: 10.1103/PhysRevLett.65.3152

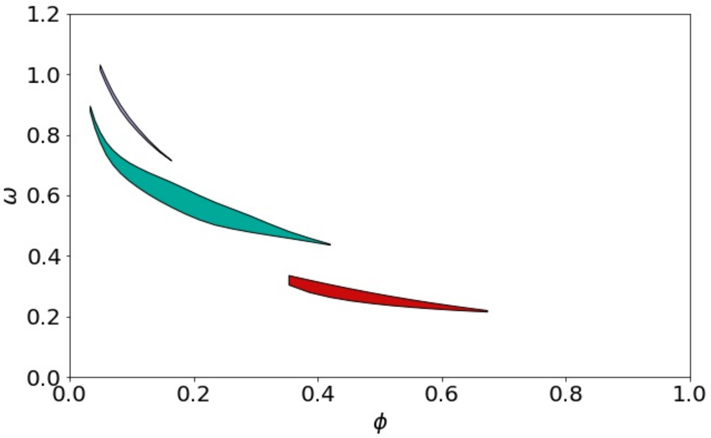

Supplementary Figure 22. Gap Atlas across filling fraction ϕ and frequency ω

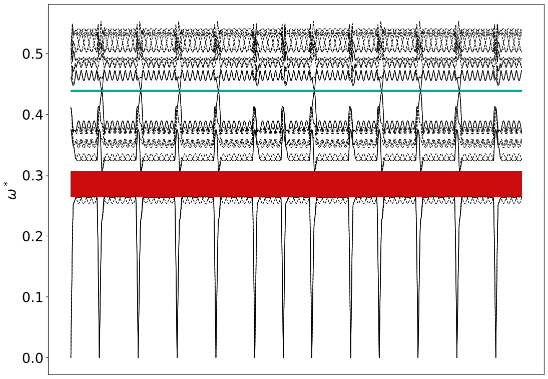

Band Structure across 1st BZ

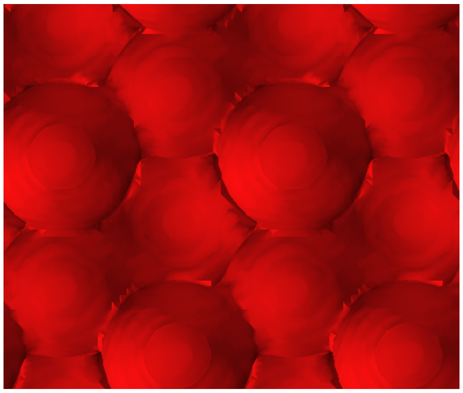

View along a_1

Supplementary Figure 23. Band Structure and Isosurface of cF8-C (Direct) at radius = 0.33, filling fraction = 0.419, where the largest gap between bands 2 and 3 occurs with gap size 14.74%.

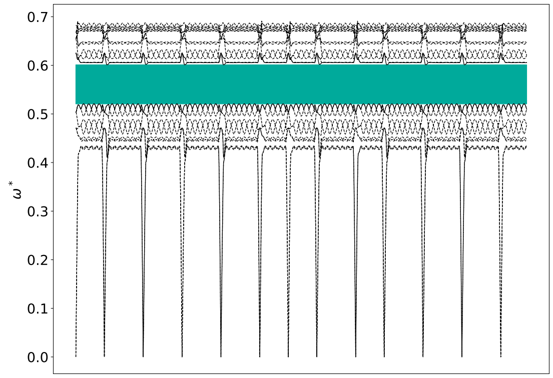

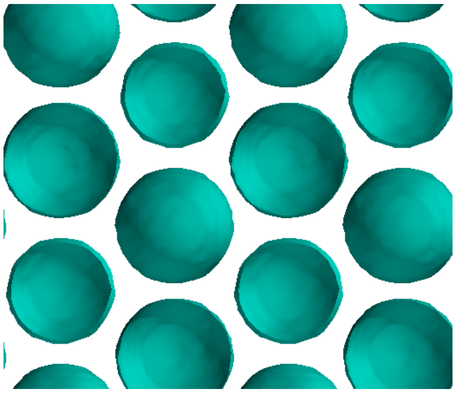

Band Structure across 1st BZ

View along a_1

Supplementary Figure 24. Band Structure and Isosurface of $cF8$-C (Direct) at radius = 0.26, filling fraction = 0.208, where the largest gap between bands 8 and 9 occurs with gap size 14.34%.

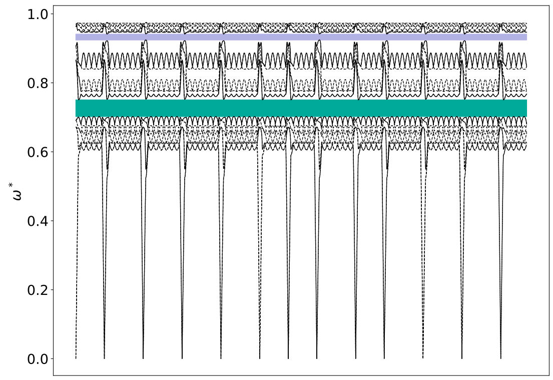

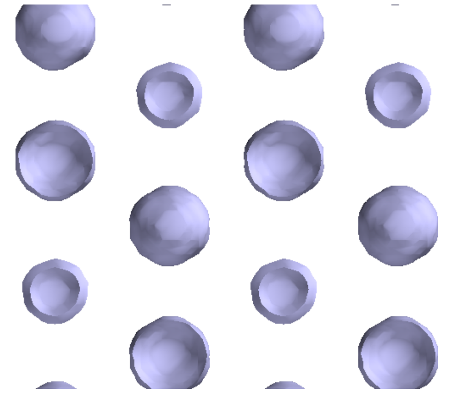

Band Structure across 1st BZ

View along a_1

Supplementary Figure 25. Band Structure and Isosurface of $cF8$-C (Direct) at radius = 0.18, filling fraction = 0.069, where the largest gap between bands 14 and 15 occurs with gap size 1.85%.

G. *cF*8-C (Inverse)

Inverse Diamond

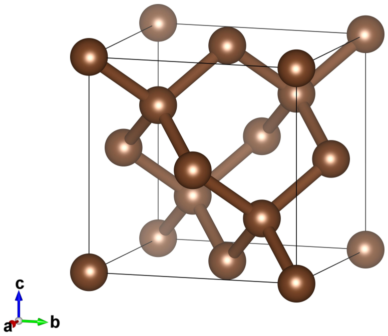

Image of $cF8$-C, generated by Vesta

$$\boldsymbol{a_1} = 1/\sqrt{2}\,\hat{y} + 1/\sqrt{2}\,\hat{z}$$
$$\boldsymbol{a_2} = 1/\sqrt{2}\,\hat{x} + 1/\sqrt{2}\,\hat{z}$$
$$\boldsymbol{a_3} = 1/\sqrt{2}\,\hat{x} + 1/\sqrt{2}\,\hat{y}$$

Space Group: 227 **Point Group:** $m\bar{3}m$

Inorganic Crystallographic Database #52054

Structure DOI: 10.1107/S0108768195010810

Photonics DOI: 10.1103/PhysRevLett.65.3152

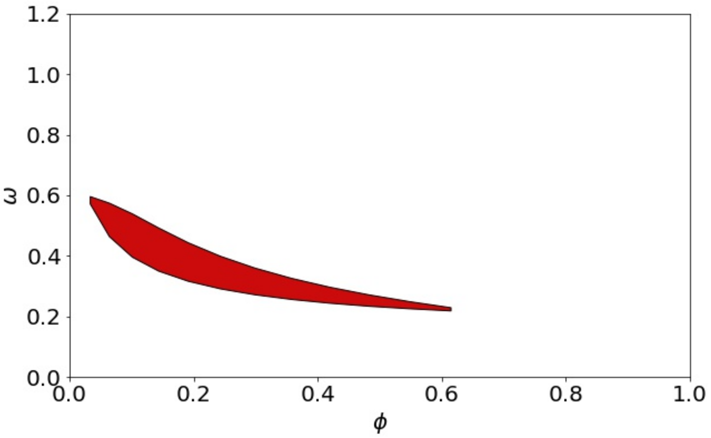

Supplementary Figure 26. Gap Atlas across filling fraction ϕ and frequency ω

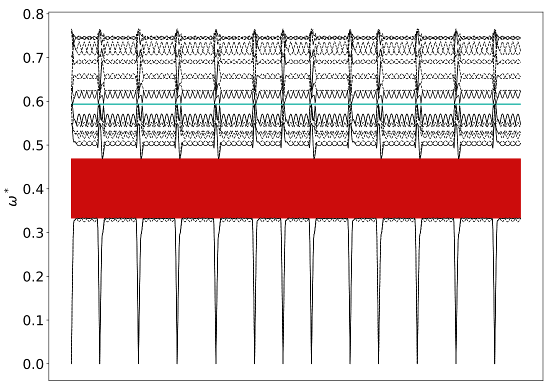

Band Structure across 1st BZ

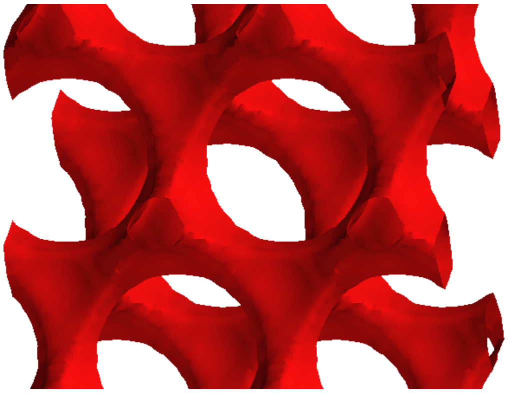

View along a_1

Supplementary Figure 27. Band Structure and Isosurface of $cF8$-C (Inverse) at radius = 0.47, filling fraction = 0.167, where the largest gap between bands 2 and 3 occurs with gap size 33.84%.

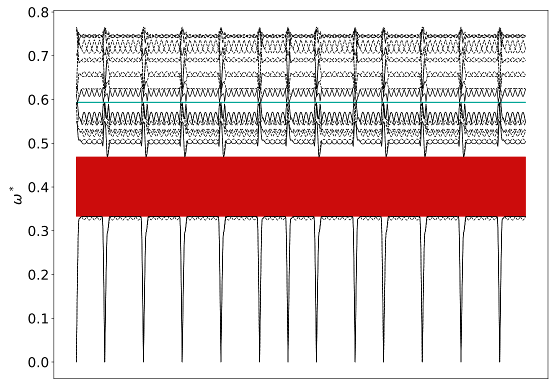

Band Structure across 1st BZ

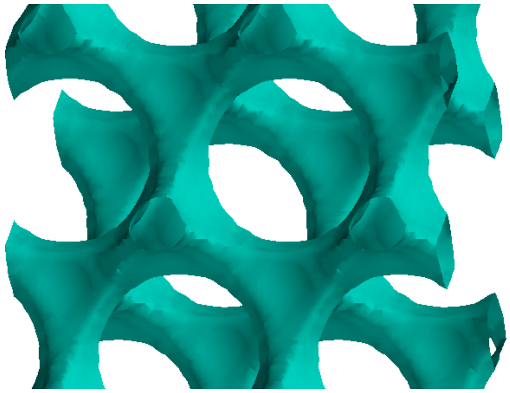

View along a_1

Supplementary Figure 28. Band Structure and Isosurface of $cF8$-C (Inverse) at radius = 0.47, filling fraction = 0.167, where the largest gap between bands 8 and 9 occurs with gap size 0.19%.

H. *cP*1-Po (Inverse)

Inverse Simple Cubic

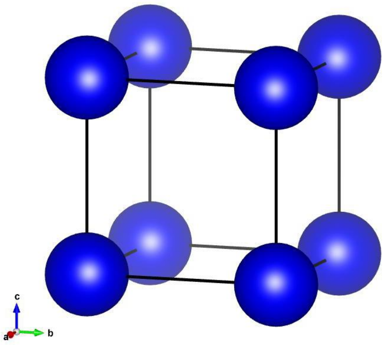

Image of *cP*1-Po, generated by Vesta

$$a_1 = \hat{x}$$
$$a_2 = \hat{y}$$
$$a_3 = \hat{z}$$

Space Group: 221 **Point Group:** $m\bar{3}m$

Inorganic Crystallographic Database #43211

Structure DOI: 10.1016/0022-1902(66)80270-1

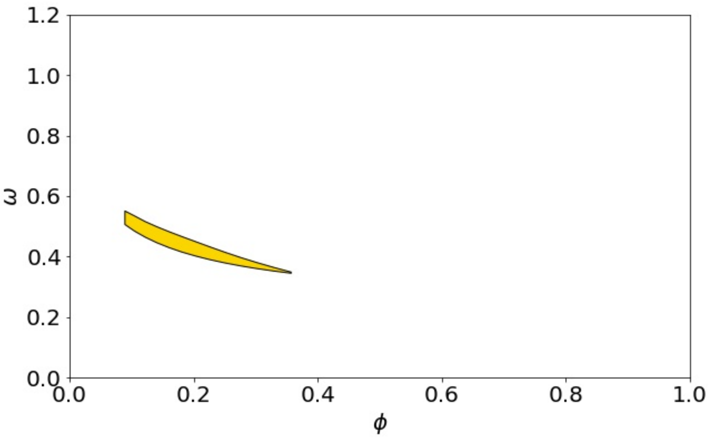

Supplementary Figure 29. Gap Atlas across filling fraction ϕ and frequency ω

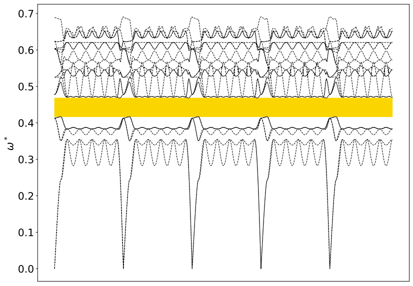

Band Structure across 1st BZ

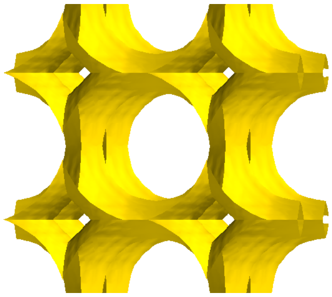

View along a_3

Supplementary Figure 30. Band Structure and Isosurface of *cP*1-Po (Inverse) at radius = 0.61, filling fraction = 0.18, where the largest gap between bands 5 and 6 occurs with gap size 11.58%.

I. *cP*24-SiO_2 (Direct)

Cristobalite (β, with defects)

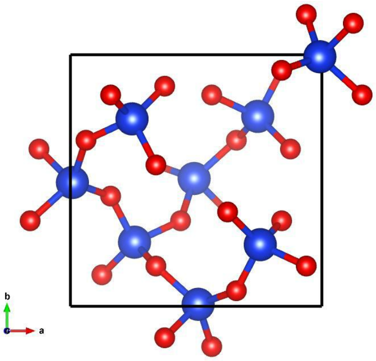

Image of *cP*24-SiO_2, generated by Vesta

$$\boldsymbol{a_1} = \hat{x}$$
$$\boldsymbol{a_2} = \hat{y}$$
$$\boldsymbol{a_3} = \hat{z}$$

Space Group: 198 **Point Group:** 23

Inorganic Crystallographic Database #24587

Structure DOI: 10.2475/ajs.s5-23.136.350

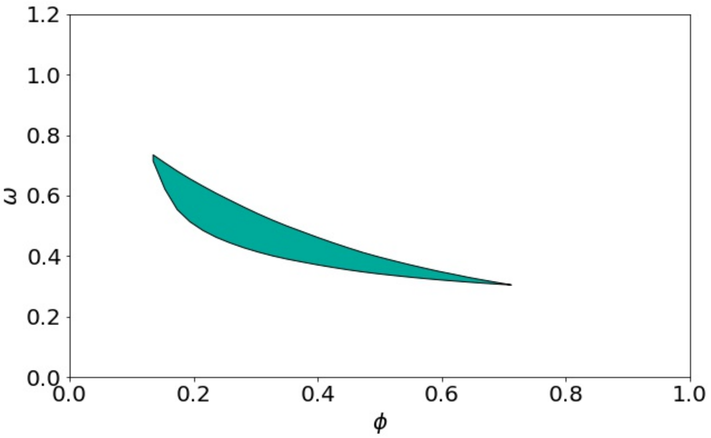

Supplementary Figure 31. Gap Atlas across filling fraction ϕ and frequency ω

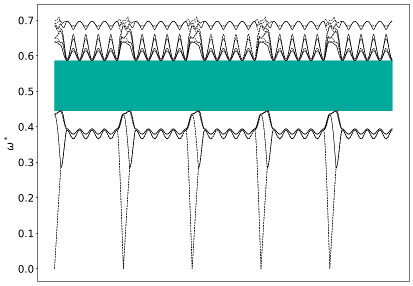

Band Structure across 1st BZ

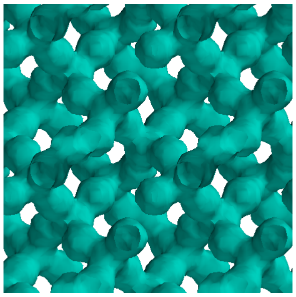

View along a_1

Supplementary Figure 32. Band Structure and Isosurface of *cP*24-SiO_2 (Direct) at radius = 0.14, filling fraction = 0.258, where the largest gap between bands 8 and 9 occurs with gap size 27.35%.

J. *cP*4-X (Inverse)

Inverse Simple Chiral Cubic

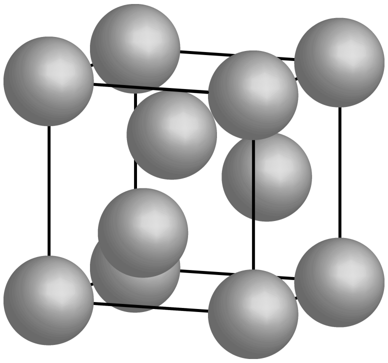

Image of *cP*4-X, generated by Vesta

$$\boldsymbol{a_1} = \hat{x}$$
$$\boldsymbol{a_2} = \hat{y}$$
$$\boldsymbol{a_3} = \hat{z}$$

Space Group: 213 **Point Group:** 432

Found in Simulation

Structure DOI: 10.1103/PhysRevLett.115.158303

Photonics DOI: 10.1063/1.1635664

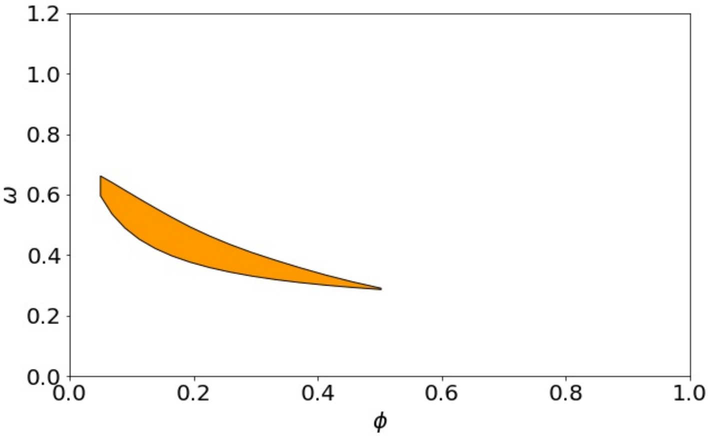

Supplementary Figure 33. Gap Atlas across filling fraction ϕ and frequency ω

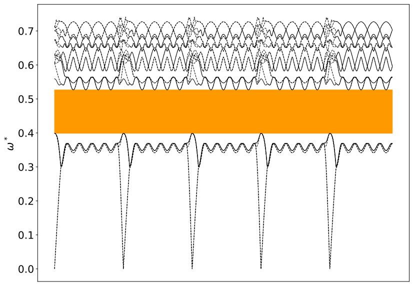

Band Structure across 1st BZ

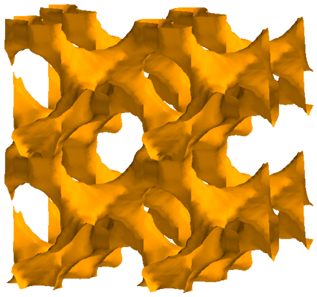

View along a_1

Supplementary Figure 34. Band Structure and Isosurface of *cP*4-X (Inverse) at radius = 0.4, filling fraction = 0.164, where the largest gap between bands 4 and 5 occurs with gap size 27.58%.

K. *oP*12-CuAsS (Direct)

Lautite

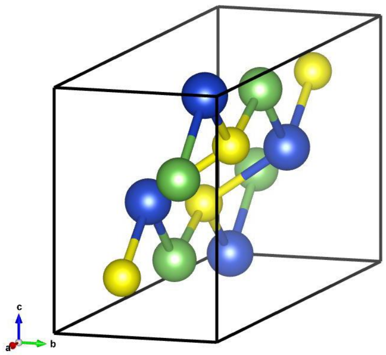

Image of *oP*12-CuAsS, generated by Vesta

$$a_1 = \hat{x}$$
$$a_2 = 0.3307746463\ \hat{y}$$
$$a_3 = 0.4805675518\ \hat{z}$$

Space Group: 62 **Point Group:** mmm

Crystallographic Open Database #2217766

Structure DOI: 10.1107/S1600536808004492

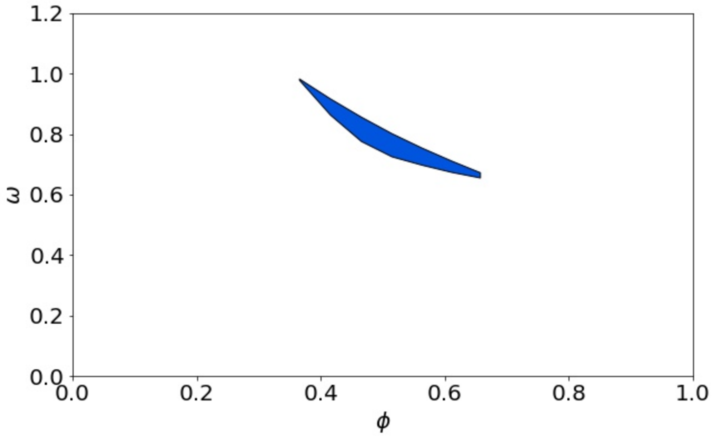

Supplementary Figure 35. Gap Atlas across filling fraction ϕ and frequency ω

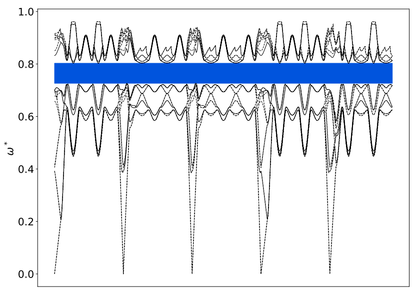

Band Structure across 1st BZ

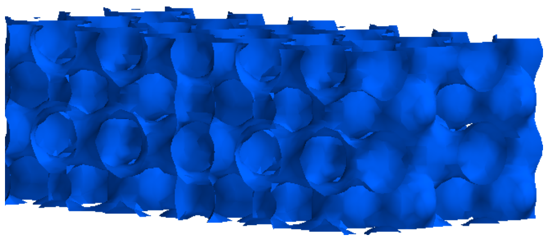

View along a_1

Supplementary Figure 36. Band Structure and Isosurface of *oP*12-CuAsS (Direct) at radius = 0.12, filling fraction = 0.514, where the largest gap between bands 12 and 13 occurs with gap size 10.0%.

L. *tI*16-$CoSiCu_2S_4$ (Direct)

Cobalt Copper Silicon Sulfide

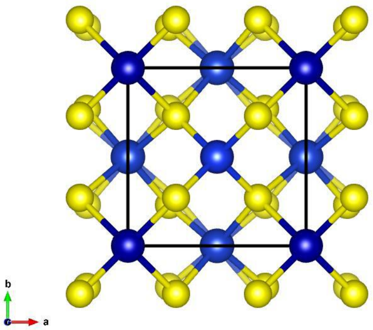

Image of *tI*16-$CoSiCu_2S_4$, generated by Vesta

$$\boldsymbol{a_1} = -0.4138049235\ \hat{x} + 0.4138049235\ \hat{y} + 0.8108828341\ \hat{z}$$
$$\boldsymbol{a_2} = 0.4138049235\ \hat{x} - 0.4138049235\ \hat{y} + 0.8108828341\ \hat{z}$$
$$\boldsymbol{a_3} = 0.4138049235\ \hat{x} + 0.4138049235\ \hat{y} - 0.8108828341\ \hat{z}$$

Space Group: 121 **Point Group:** $\bar{4}2m$

Crystallographic Open Database #1533601

Structure DOI: 10.1016/j.jallcom.2004.02.004

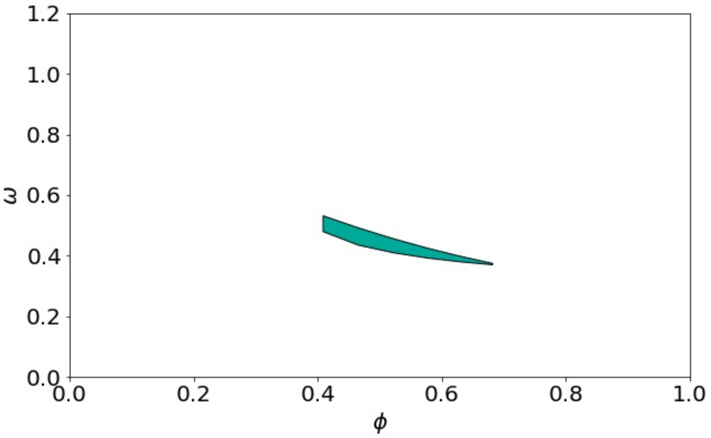

Supplementary Figure 37. Gap Atlas across filling fraction ϕ and frequency ω

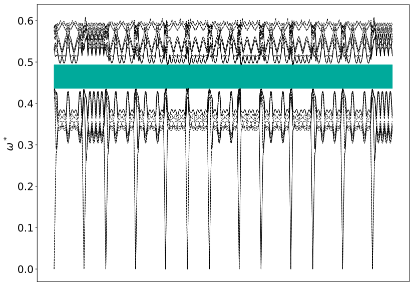

Band Structure across 1st BZ

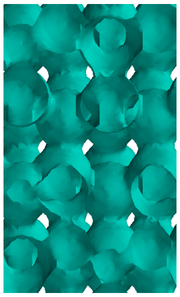

View along a_1

Supplementary Figure 38. Band Structure and Isosurface of *tI*16-$CoSiCu_2S_4$ (Direct) at radius = 0.2, filling fraction = 0.465, where the largest gap between bands 8 and 9 occurs with gap size 12.26%.

M. *tI*8-YMn_2 (Direct)

Manganese Yttrium

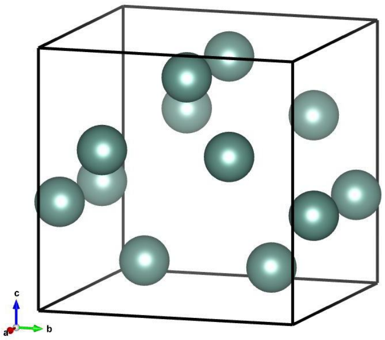

Image of *tI*8-YMn_2, generated by Vesta

$$a_1 = -0.5783430911\ \hat{x} + 0.5783430911\ \hat{y} + 0.5753594859\ \hat{z}$$
$$a_2 = 0.5783430911\ \hat{x} - 0.5783430911\ \hat{y} + 0.5753594859\ \hat{z}$$
$$a_3 = 0.5783430911\ \hat{x} + 0.5783430911\ \hat{y} - 0.5753594859\ \hat{z}$$

Space Group: 141 **Point Group:** $4/mmm$

Crystallographic Open Database #1524241

Structure DOI: 10.1088/0953-8984/3/33/023

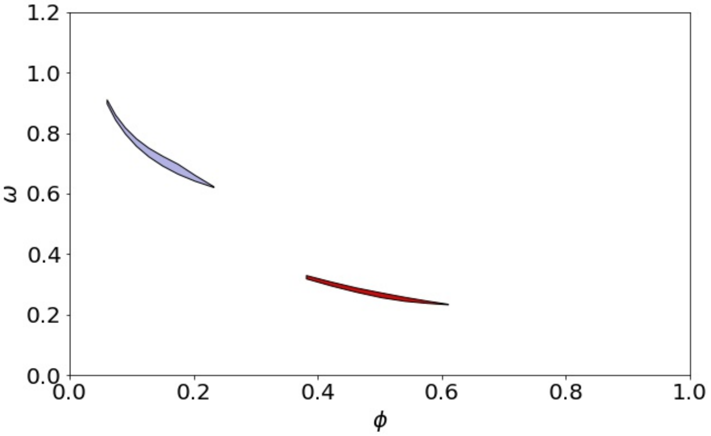

Supplementary Figure 39. Gap Atlas across filling fraction ϕ and frequency ω

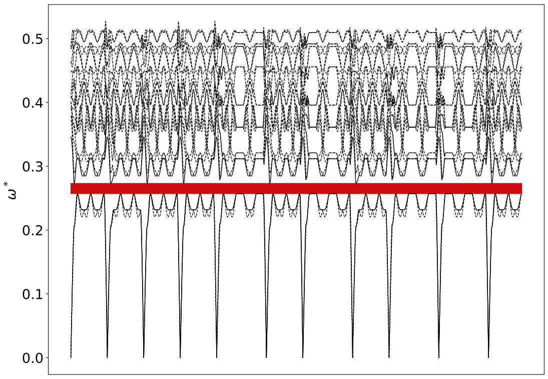

Band Structure across 1st BZ

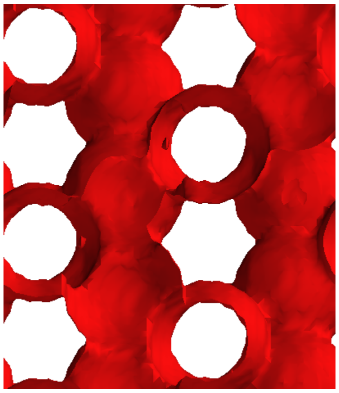

View rotated 45° about l_3 and elevated 0○

Supplementary Figure 40. Band Structure and Isosurface of *tI*8-YMn_2 (Direct) at radius = 0.29, filling fraction = 0.502, where the largest gap between bands 2 and 3 occurs with gap size 6.01%.

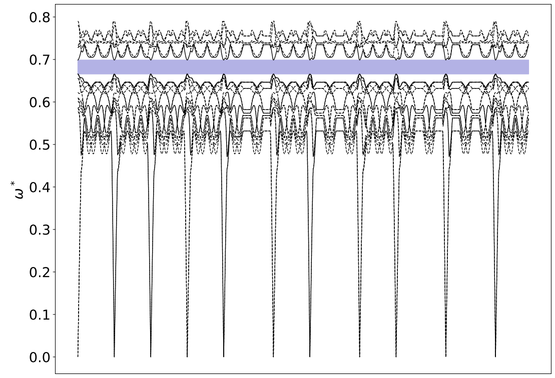

Band Structure across 1st BZ

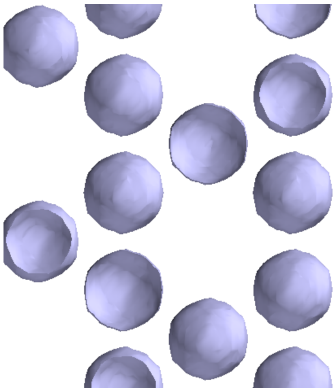

View along a_1

Supplementary Figure 41. Band Structure and Isosurface of *tI*8-YMn_2 (Direct) at radius = 0.2, filling fraction = 0.174, where the largest gap between bands 14 and 15 occurs with gap size 4.82%.

N. *tP*12-SiO_2 (Direct)

Cristobalite (α, HT)

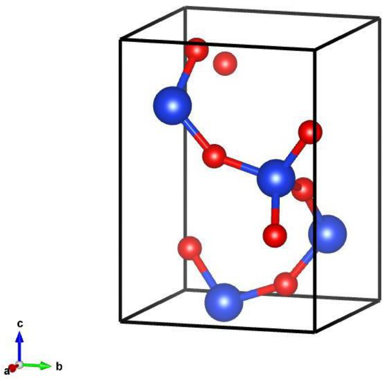

Image of *tP*12-SiO_2, generated by Vesta

$$a_1 = \hat{x}$$
$$a_2 = \hat{y}$$
$$a_3 = 1.3977078859161431\ \hat{z}$$

Space Group: 92 **Point Group:** 422

Inorganic Crystallographic Database #34928

Structure DOI: 10.1524/zkri.1973.138.jg.274

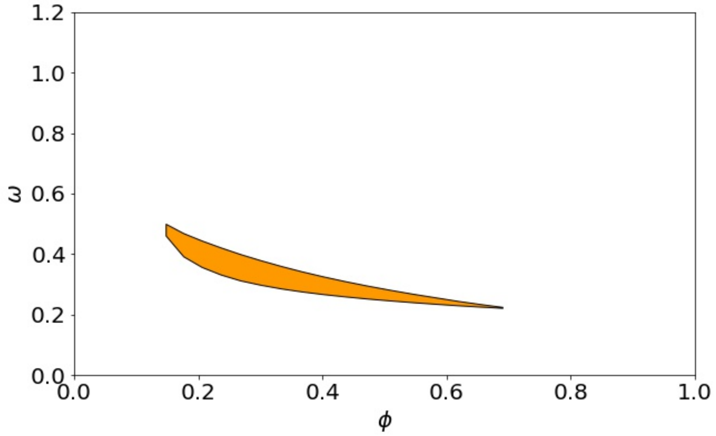

Supplementary Figure 42. Gap Atlas across filling fraction ϕ and frequency ω

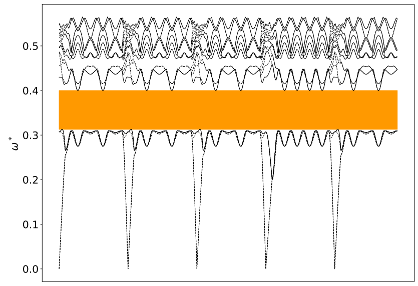

Band Structure across 1st BZ

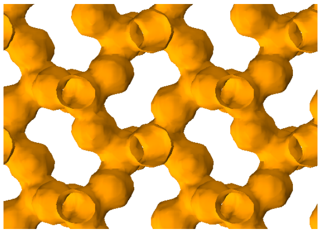

View along a_2

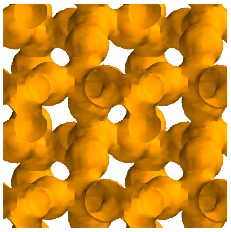

View along a_1

Supplementary Figure 43. Band Structure and Isosurface of *tP*12-SiO_2 (Direct) at radius = 0.2, filling fraction = 0.036, where the largest gap between bands 4 and 5 occurs with gap size 24.44%.

O. *tP*4-PdO (Direct)

Palladium Oxide

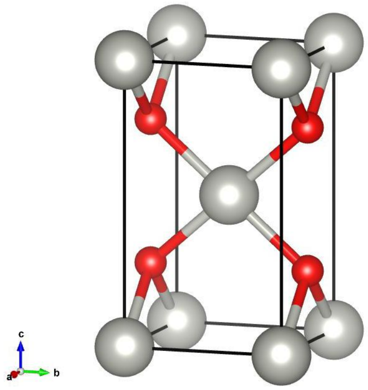

Image of *tP*4-PdO, generated by Vesta

$\boldsymbol{a_1} = \ \hat{x}$
$\boldsymbol{a_2} = \ \hat{y}$
$\boldsymbol{a_3} = 1.759075899\ \hat{z}$

Space Group: 131 **Point Group:** $4/mmm$

Crystallographic Open Database #1009031

Structure DOI: 10.1107/S0365110X53001800

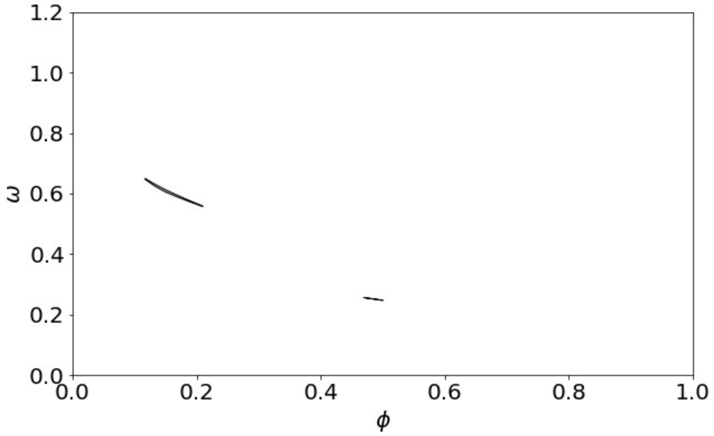

Supplementary Figure 44. Gap Atlas across filling fraction ϕ and frequency ω

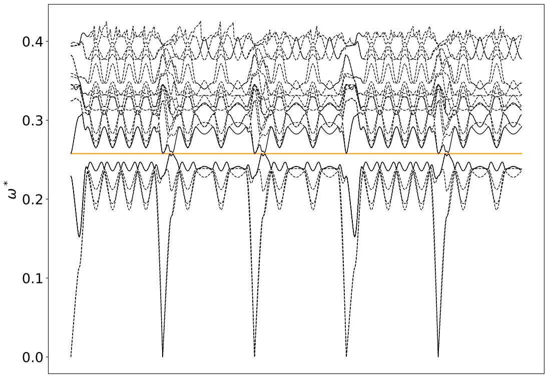

Band Structure across 1st BZ

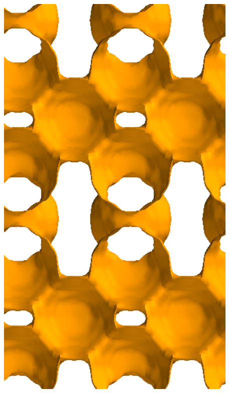

View along a_1

Supplementary Figure 45. Band Structure and Isosurface of *tP*4-PdO (Direct) at radius = 0.37, filling fraction = 0.621, where the largest gap between bands 4 and 5 occurs with gap size 0.18%.

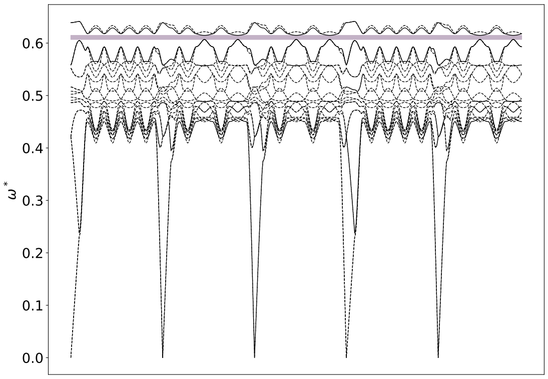

Band Structure across 1st BZ

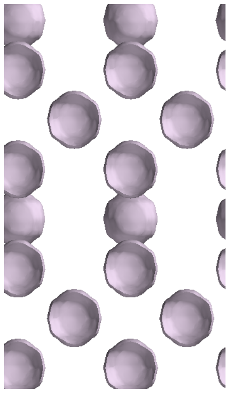

View along a_1

Supplementary Figure 46. Band Structure and Isosurface of *tP*4-PdO (Direct) at radius = 0.25, filling fraction = 0.257, where the largest gap between bands 18 and 19 occurs with gap size 1.28%.

P. *hP*21-$NaAlSiO_4$ (Direct)

Sodium Alumosilicate Eu-doped

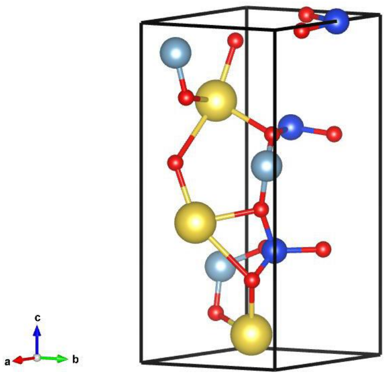

Image of *hP*21-$NaAlSiO_4$, generated by Vesta

$$\boldsymbol{a_1} = \hat{x}$$
$$\boldsymbol{a_2} = -1/2\,\hat{x} + \sqrt{3/4}\,\hat{y}$$
$$\boldsymbol{a_3} = 2.444235794\,\hat{z}$$

Space Group: 145 **Point Group:** 3

Inorganic Crystallographic Database #433181

Structure DOI: 10.1021/acs.chemmater.7b02548

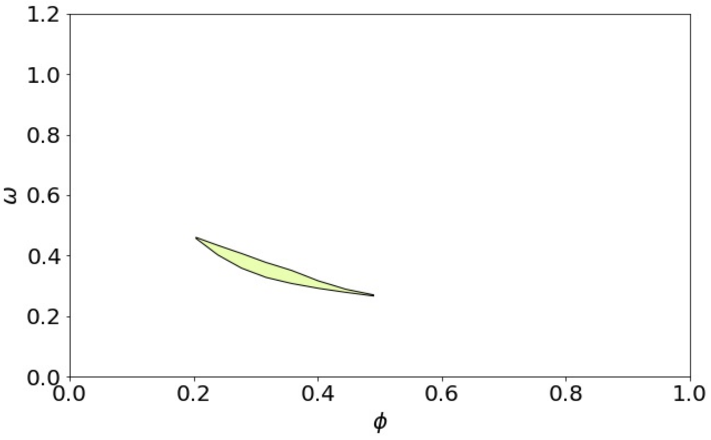

Supplementary Figure 47. Gap Atlas across filling fraction ϕ and frequency ω

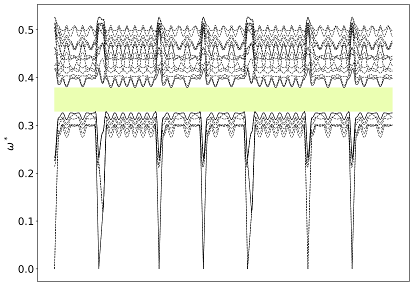

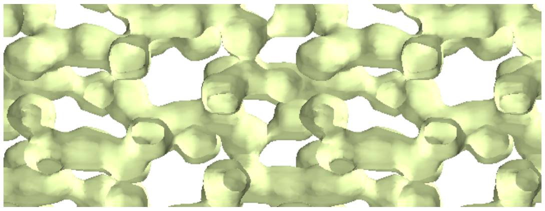

Band Structure across 1st BZ

Supplementary Figure 48. Band Structure and Isosurface of *hP*21-$NaAlSiO_4$ (Direct) at radius = 0.2, filling fraction = 0.316, where the largest gap between bands 6 and 7 occurs with gap size 13.97%.

Q. *cP*26-UBe$_{13}$ (Inverse)

Inverse AB_{13}

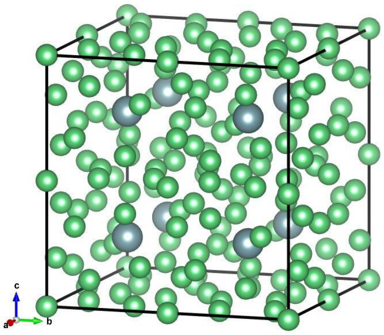

Image of *cP*26-UBe$_{13}$, generated by Vesta

$\boldsymbol{a_1} = \hat{x}$
$\boldsymbol{a_2} = \hat{y}$
$\boldsymbol{a_3} = \hat{z}$

Space Group: 221 **Point Group:** $m\bar{3}m$

Inorganic Crystallographic Database #58751

Structure DOI: 10.1103/PhysRevB.32.6042

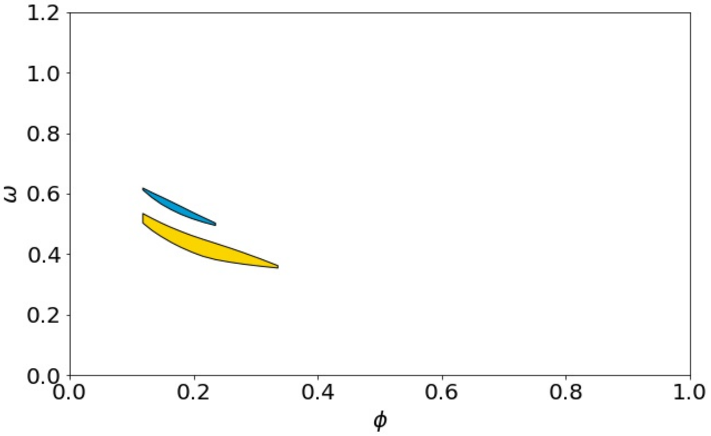

Supplementary Figure 49. Gap Atlas across filling fraction ϕ and frequency ω

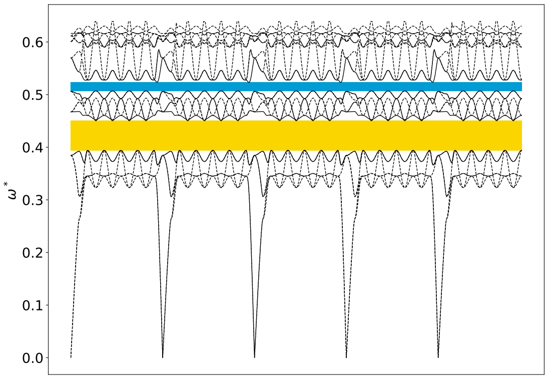

Band Structure across 1st BZ

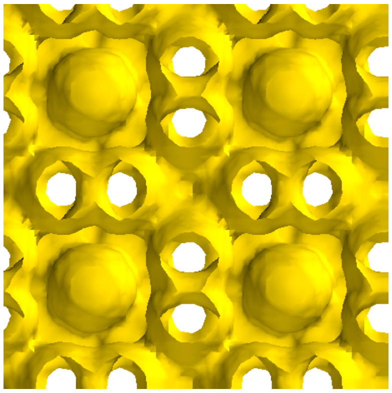

View along a_1

Supplementary Figure 50. Band Structure and Isosurface of *cP*26-UBe$_{13}$ (Inverse) at radius = 0.23, filling fraction = 0.214, where the largest gap between bands 5 and 6 occurs with gap size 13.3%.

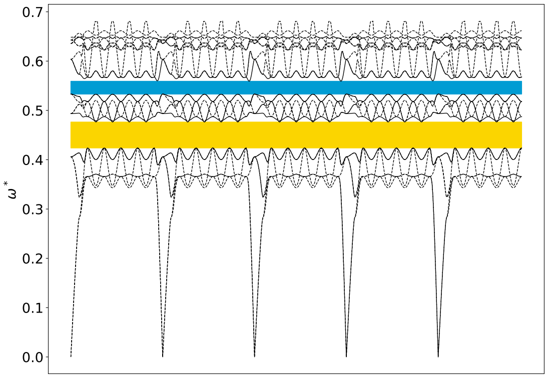

Band Structure across 1st BZ

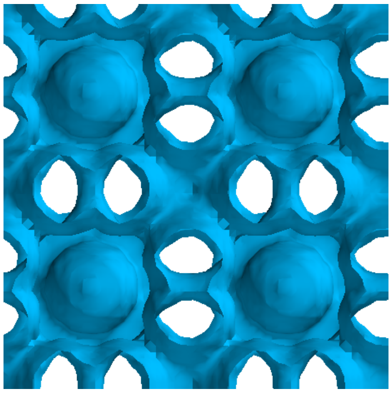

View along a_1

Supplementary Figure 51. Band Structure and Isosurface of *cP*26-UBe_{13} (Inverse) at radius = 0.24, filling fraction = 0.179, where the largest gap between bands 10 and 11 occurs with gap size 4.77%.

R. *mP*24-SiO_2 (Inverse)

Inverse Monoclinic Cristobalite (II)

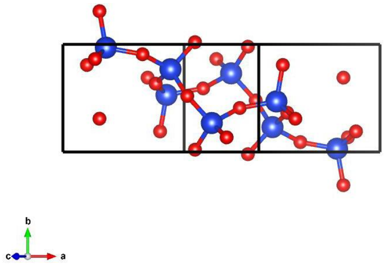

$$\boldsymbol{a_1} = \hat{x}$$
$$\boldsymbol{a_2} = 0.5493679512424072\,\hat{y}$$
$$\boldsymbol{a_3} = -0.6192893815130248\,\hat{x} + 0.8864439445037495\,\hat{z}$$

Image of *mP*24-SiO_2, generated by Vesta

Space Group: 14 **Point Group:** $2/m$

Inorganic Crystallographic Database #91737

Structure DOI: 10.1180/002646100549436

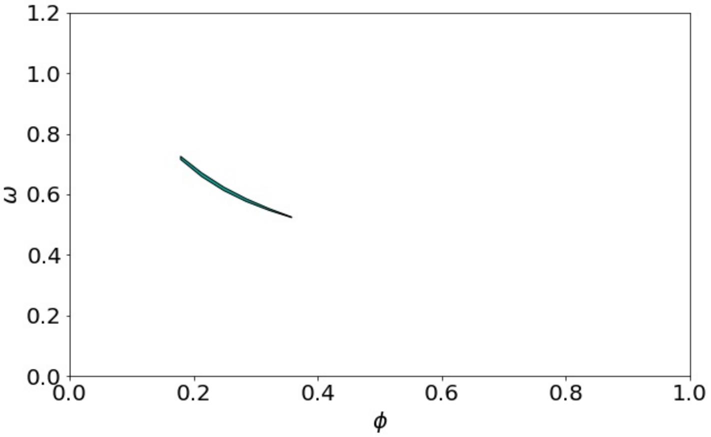

Supplementary Figure 52. Gap Atlas across filling fraction ϕ and frequency ω

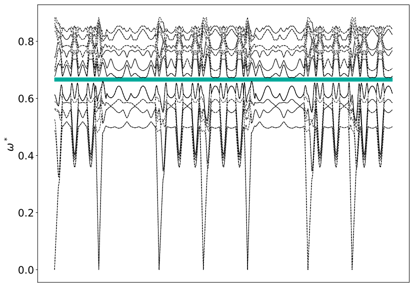

Band Structure across 1st BZ

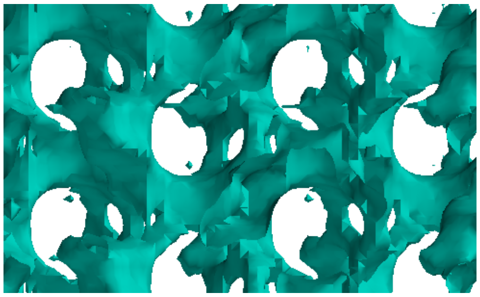

View along a_1

Supplementary Figure 53. Band Structure and Isosurface of *mP*24-SiO_2 (Inverse) at radius = 0.185, filling fraction = 0.212, where the largest gap between bands 8 and 9 occurs with gap size 1.88%.

S. *oF*8-Pu (Inverse)

Inverse Plutonium (γ)

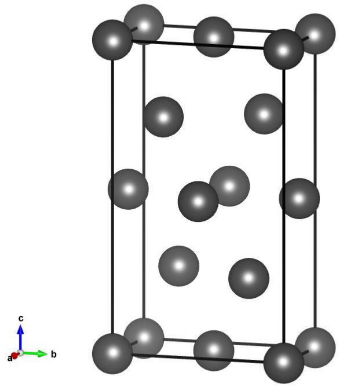

Image of $oF8$-Pu, generated by Vesta

$$\boldsymbol{a_1} = 0.4936429625\ \hat{y} + 0.8696646627\ \hat{z}$$
$$\boldsymbol{a_2} = 0.2703217714\ \hat{x} + 0.8696646627\ \hat{z}$$
$$\boldsymbol{a_3} = 0.2703217714\ \hat{x} + 0.4936429625\ \hat{y}$$

Space Group: 70 **Point Group:** mmm

Inorganic Crystallographic Database #44866

Structure DOI: 10.1107/S0365110X55001357

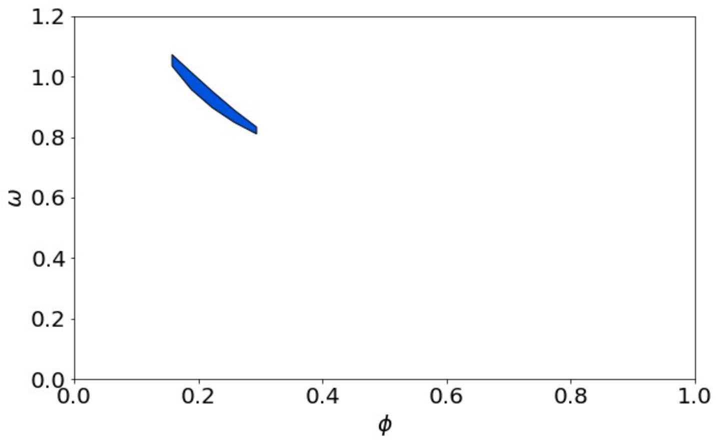

Supplementary Figure 54. Gap Atlas across filling fraction ϕ and frequency ω

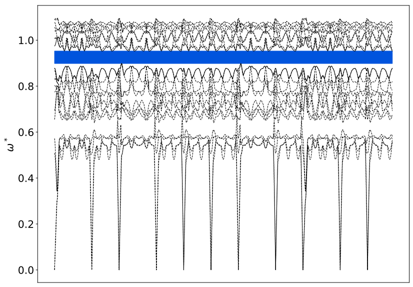

Band Structure across 1st BZ

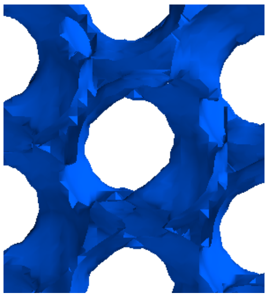

View along a_1

Supplementary Figure 55. Band Structure and Isosurface of $oF8$-Pu (Inverse) at radius = 0.28, filling fraction = 0.222, where the largest gap between bands 12 and 13 occurs with gap size 5.87%.

T. *mC*64-P_3N_5 (Direct)

Phosphorus (V) Nitride (α)

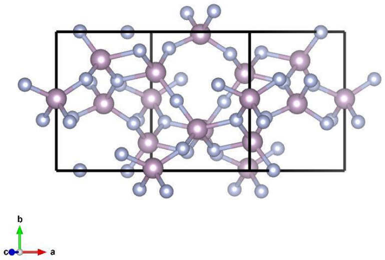

Image of *mC*64-P_3N_5, generated by Vesta

$$\boldsymbol{a_1} = \hat{x}$$
$$\boldsymbol{a_2} = 0.7184453882\,\hat{y}$$
$$\boldsymbol{a_3} = -0.4910905848\,\hat{x} + 1.0154626529\,\hat{z}$$

Space Group: 9 **Point Group:** m

Crystallographic Open Database #6000643

Structure DOI: 10.1002/(SICI)1521-3749(199804)624:4<620::AID-ZAAC620>3.0.CO;2-K

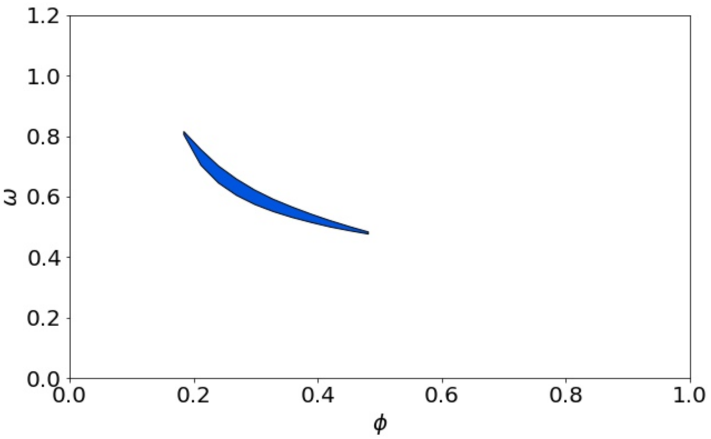

Supplementary Figure 56. Gap Atlas across filling fraction ϕ and frequency ω

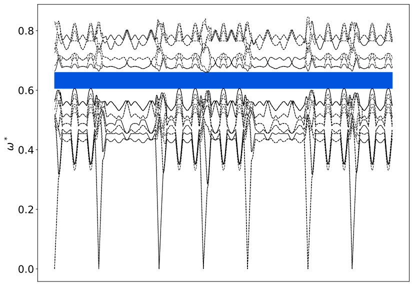

Band Structure across 1st BZ

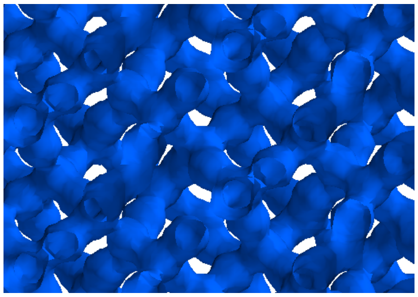

View along a_1

Supplementary Figure 57. Band Structure and Isosurface of *mC*64-P_3N_5 (Direct) at radius = 0.115, filling fraction = 0.158, where the largest gap between bands 12 and 13 occurs with gap size 8.53%.

U. *cI*16-Si (Inverse)

Inverse Silicon (II)

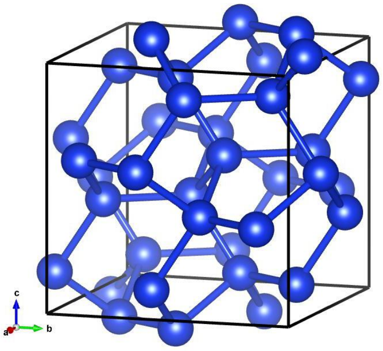

Image of *cI*16-Si, generated by Vesta

$$\boldsymbol{a_1} = -1/\sqrt{3}\,\hat{x} + 1/\sqrt{3}\,\hat{y} + 1/\sqrt{3}\,\hat{z}$$
$$\boldsymbol{a_2} = 1/\sqrt{3}\,\hat{x} - 1/\sqrt{3}\,\hat{y} + 1/\sqrt{3}\,\hat{z}$$
$$\boldsymbol{a_3} = 1/\sqrt{3}\,\hat{x} + 1/\sqrt{3}\,\hat{y} - 1/\sqrt{3}\,\hat{z}$$

Space Group: 206 **Point Group:** $m\bar{3}$

Inorganic Crystallographic Database #16569

Structure DOI: 10.1107/S0365110X64001840

Photonics DOI: 10.1134/S0021364007160047

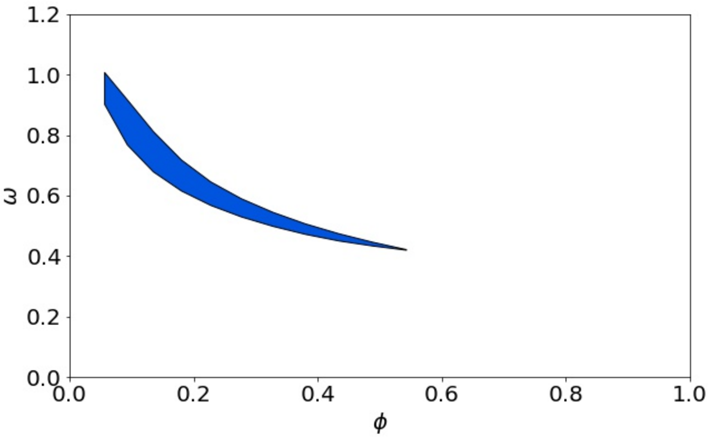

Supplementary Figure 58. Gap Atlas across filling fraction ϕ and frequency ω

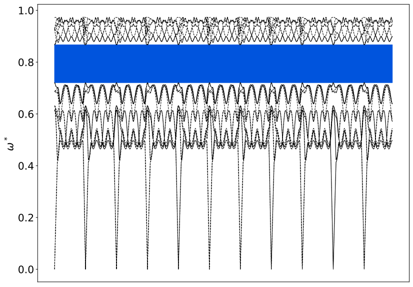

Band Structure across 1st BZ

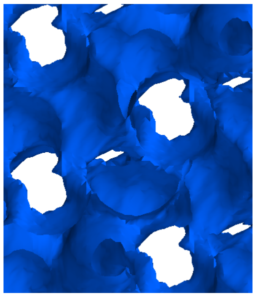

View along a_1

Supplementary Figure 59. Band Structure and Isosurface of *cI*16-Si (Inverse) at radius = 0.305, filling fraction = 0.292, where the largest gap between bands 12 and 13 occurs with gap size 18.44%.

V. *mC*4-Ce (Inverse)

Inverse Cerium (α, HP)

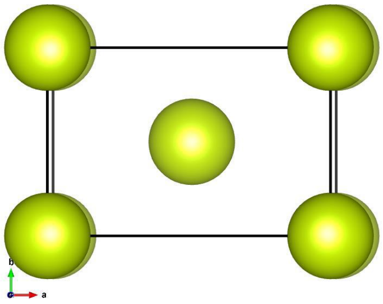

Image of *mC*4-Ce, generated by Vesta

$\boldsymbol{a_1} = \hat{x}$
$\boldsymbol{a_2} = 0.6656866906\,\hat{y}$
$\boldsymbol{a_3} = -0.0200905187\,\hat{x} + 0.6651733278\,\hat{z}$

Space Group: 12 **Point Group:** $2/m$

Inorganic Crystallographic Database #41824

Structure DOI: 10.1107/S0567739477000321

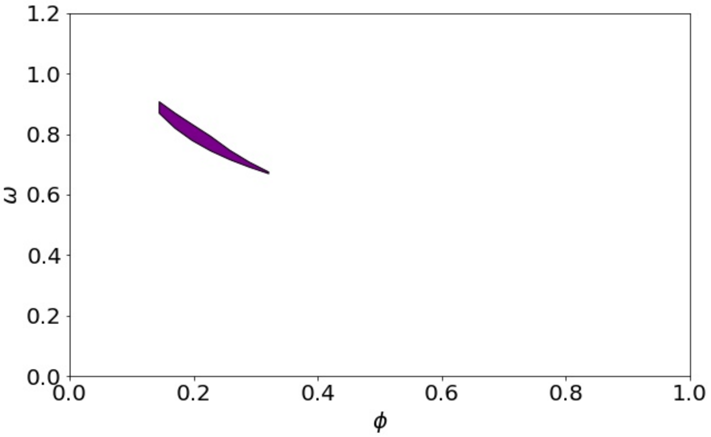

Supplementary Figure 60. Gap Atlas across filling fraction ϕ and frequency ω

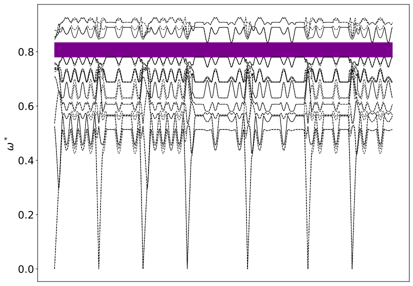

Band Structure across 1st BZ

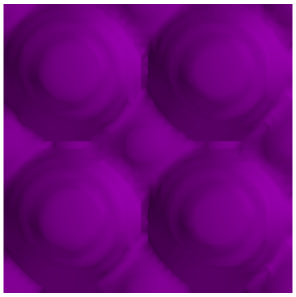

View along a_1

Supplementary Figure 61. Band Structure and Isosurface of *mC*4-Ce (Inverse) at radius = 0.35, filling fraction = 0.197, where the largest gap between bands 16 and 17 occurs with gap size 6.62%.

W. *hP*4-Ge (Direct)

Hexagonal Diamond

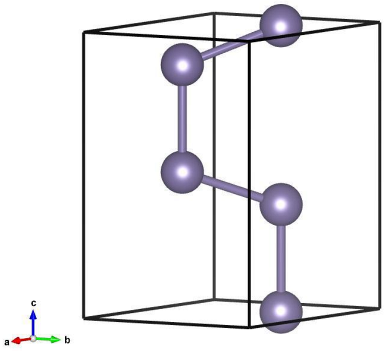

Image of *hP*4-Ge, generated by Vesta

$\boldsymbol{a_1} = \hat{x}$
$\boldsymbol{a_2} = -1/2\,\hat{x} + \sqrt{3/4}\,\hat{y}$
$\boldsymbol{a_3} = 1.6624365725\,\hat{z}$

Space Group: 194 **Point Group:** $6/mmm$

Inorganic Crystallographic Database #636533

Structure DOI: 10.17188/1324726

Photonics DOI: 10.1088/0953-8984/16/6/005

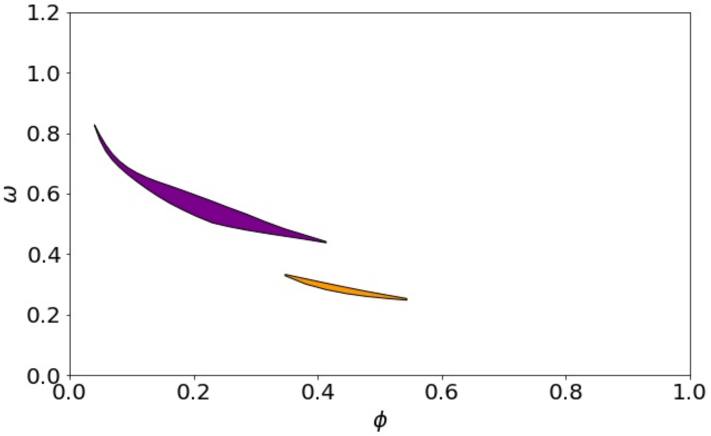

Supplementary Figure 62. Gap Atlas across filling fraction ϕ and frequency ω

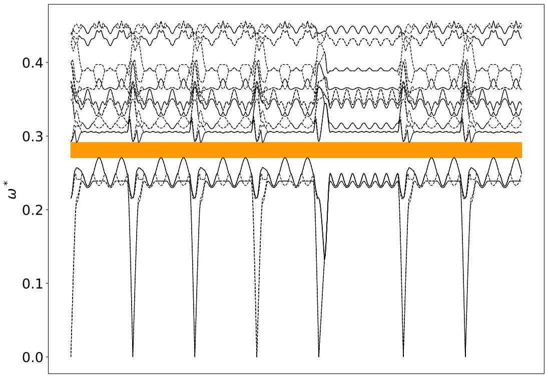

Band Structure across 1st BZ

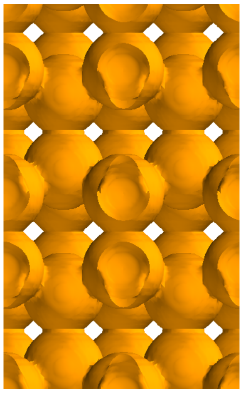

View along a_1

Supplementary Figure 63. Band Structure and Isosurface of *hP*4-Ge (Direct) at radius = 0.34, filling fraction = 0.161, where the largest gap between bands 4 and 5 occurs with gap size 7.33%.

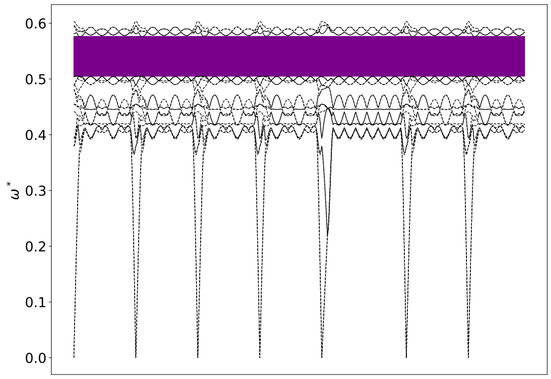

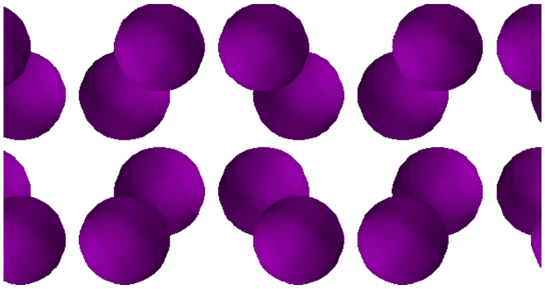

View along a_1

Band Structure across 1st BZ

Supplementary Figure 64. Band Structure and Isosurface of *hP*4-Ge (Direct) at radius = 0.27, filling fraction = 0.057, where the largest gap between bands 16 and 17 occurs with gap size 13.2%.

X. *hR*1-Po (Inverse)

Inverse Polonium (β)

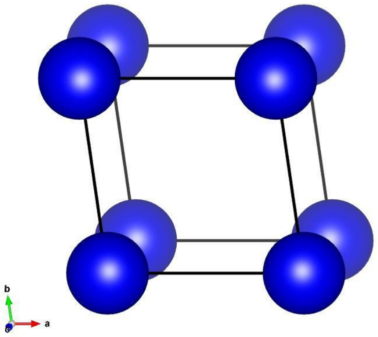

Image of *hR*1-Po, generated by Vesta

$$\boldsymbol{a_1} = \hat{x}$$
$$\boldsymbol{a_2} = -0.1433198562\,\hat{x} + 0.9896764213\,\hat{y}$$
$$\boldsymbol{a_3} = -0.1433198562\,\hat{x} - 0.1655697092\,\hat{y} + 0.9757284921\,\hat{z}$$

Space Group: 166 **Point Group:** $\bar{3}m$

Inorganic Crystallographic Database #43212

Structure DOI: 10.1016/0022-1902(66)80270-1

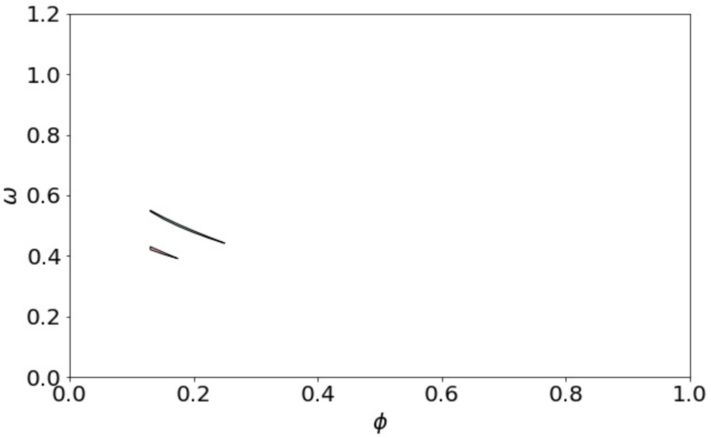

Supplementary Figure 65. Gap Atlas across filling fraction ϕ and frequency ω

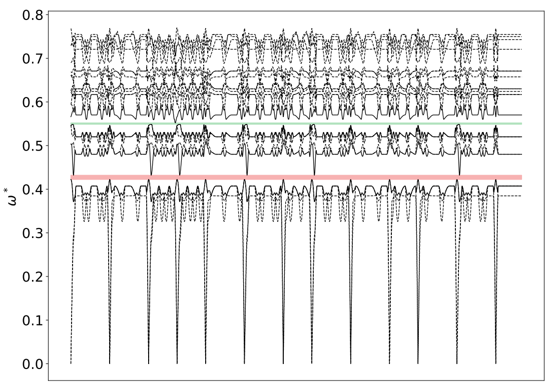

Band Structure across 1st BZ

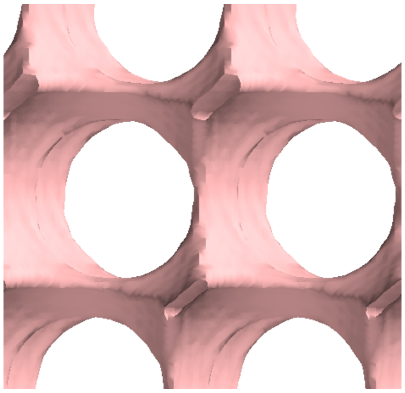

View along a_1

Supplementary Figure 66. Band Structure and Isosurface of *hR*1-Po (Inverse) at radius = 0.62, filling fraction = 0.129, where the largest gap between bands 3 and 4 occurs with gap size 2.28%.

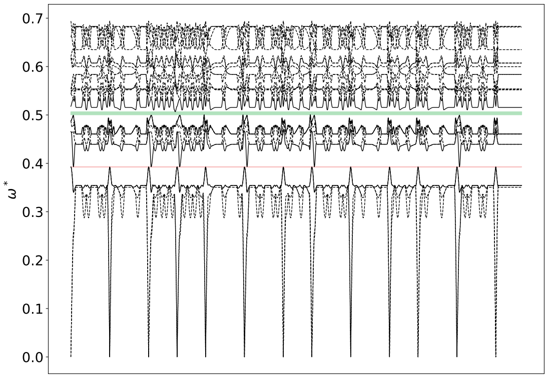

Band Structure across 1st BZ

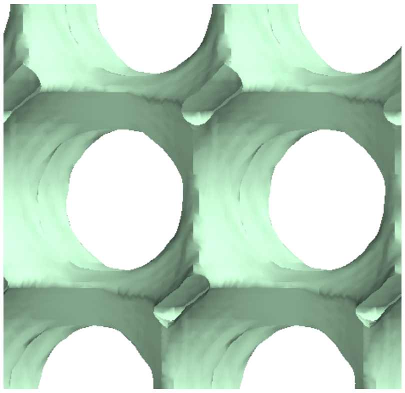

View along a_1

Supplementary Figure 67. Band Structure and Isosurface of *hR*1-Po (Inverse) at radius = 0.6, filling fraction = 0.174, where the largest gap between bands 7 and 8 occurs with gap size 1.25%.

Y. $oC48$-H_2O (Direct)

Ice II

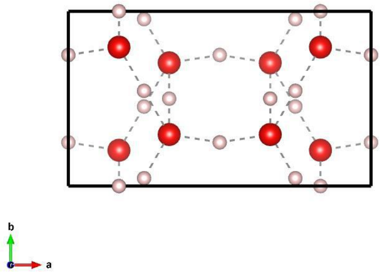

Image of $oC48$-H_2O, generated by Vesta

$$\mathbf{a_1} = \hat{x}$$
$$\mathbf{a_2} = 0.5769230628\,\hat{y}$$
$$\mathbf{a_3} = 0.7128204881\,\hat{z}$$

Space Group: 20 **Point Group:** 222

Crystallographic Open Database #1011063

Structure DOI: 10.1063/1.1749748

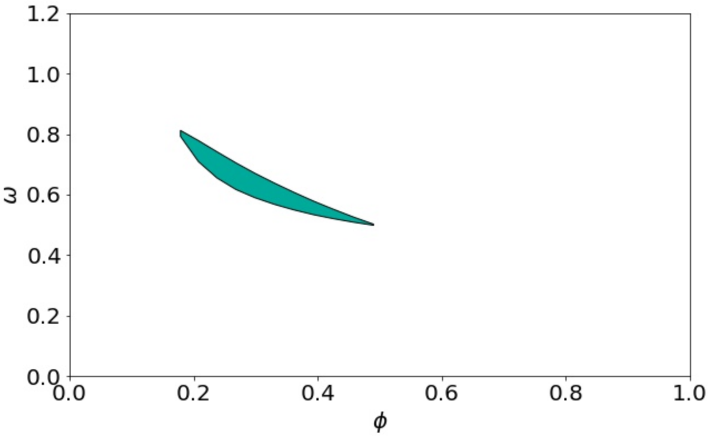

Supplementary Figure 68. Gap Atlas across filling fraction ϕ and frequency ω

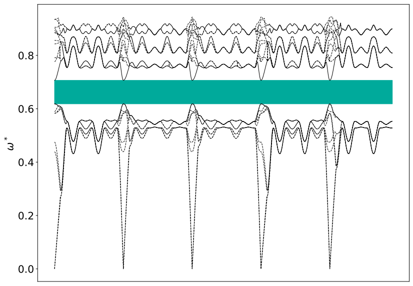

Band Structure across 1st BZ

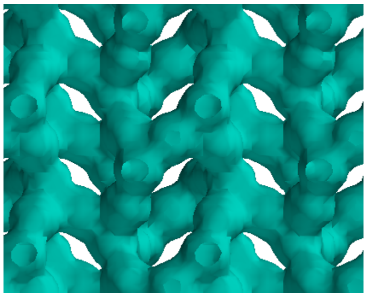

View along a_1

Supplementary Figure 69. Band Structure and Isosurface of $oC48$-H_2O (Direct) at radius = 0.105, filling fraction = 0.237, where the largest gap between bands 8 and 9 occurs with gap size 13.3%.

Z. *hP*20-H_3O (Inverse)

Inverse Ice Ih

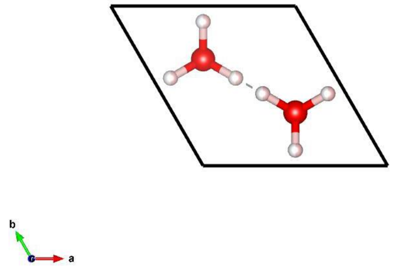

$$a_1 = \hat{x}$$
$$a_2 = -1/2\,\hat{x} + \sqrt{3/4}\,\hat{y}$$
$$a_3 = 1.6281081102\,\hat{z}$$

Image of *hP*20-H_3O, generated by Vesta

Space Group: 194 **Point Group:** $6/mmm$

Inorganic Crystallographic Database #247095

Structure DOI: 10.1063/1.1765099

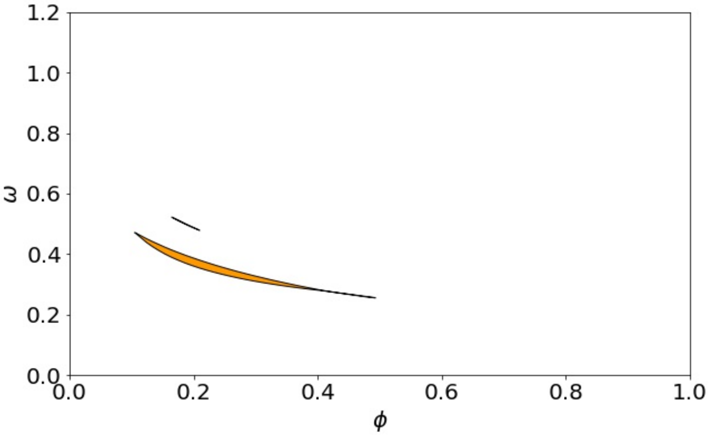

Supplementary Figure 70. Gap Atlas across filling fraction ϕ and frequency ω

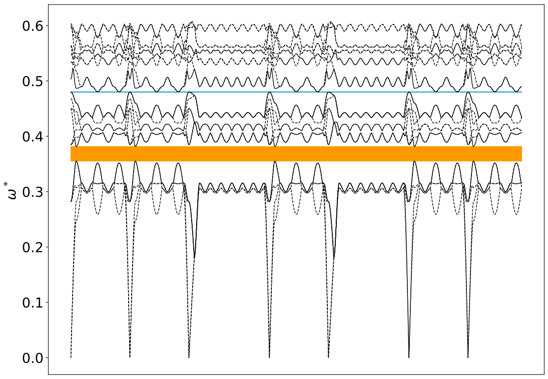

Band Structure across 1st BZ

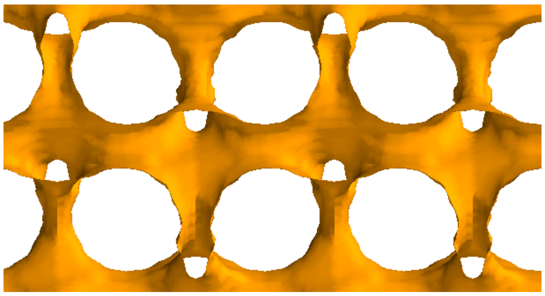

View along a_1

Supplementary Figure 71. Band Structure and Isosurface of *hP*20-H_3O (Inverse) at radius = 0.375, filling fraction = 0.209, where the largest gap between bands 4 and 5 occurs with gap size 7.08%.

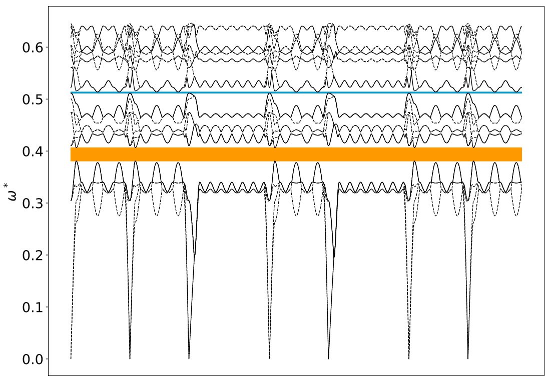

Band Structure across 1st BZ

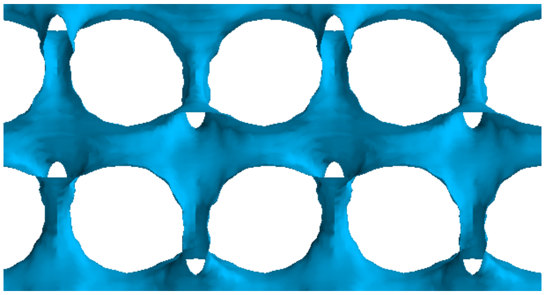

View along a_1

Supplementary Figure 72. Band Structure and Isosurface of *hP*20-H_3O (Inverse) at radius = 0.39, filling fraction = 0.175, where the largest gap between bands 10 and 11 occurs with gap size 0.41%.

SUPPLEMENTARY REFERENCES

[1]Ho, K. M., Chan, C. T. & Soukoulis, C. M. Existence of a photonic gap in periodic dielectric structures. *Physical Review Letters* **65**, 3152–3155 (1990). URL http://www.ncbi.nlm.nih.gov/pubmed/10042794https://link.aps.org/doi/10.1103/PhysRevLett.65.3152.

[2]Sözüer, H. S. & Haus, J. W. Photonic bands: simple-cubic lattice. *Journal of the Optical Society of America B* **10**, 296 (1993). URL https://www.osapublishing.org/abstract.cfm?URI=josab-10-2-296.

[3]Chen, H., Zhang, W. & Wang, Z. Comparative studies on photonic band structures of diamond and hexagonal diamond using the multiple scattering method. *Journal of Physics: Condensed Matter* **16**, 741–748 (2004). URL http://stacks.iop.org/0953-8984/16/i=6/a=005?key=crossref.9ec19323ea3e02086f8d120bd666e276.

[4]Johnson, S. & Joannopoulos, J. Block-iterative frequency-domain methods for Maxwell's equations in a planewave basis. *Optics Express* **8**, 173 (2001).

[5]Sözüer, H. S., Haus, J. W. & Inguva, R. Photonic bands: Convergence problems with the plane-wave method. *Physical Review B* **45**, 13962–13972 (1992). URL https://journals.aps.org/prb/pdf/10.1103/PhysRevB.45.13962https://link.aps.org/doi/10.1103/PhysRevB.45.13962.

[6]Moroz, A. Three-Dimensional Complete Photonic-Band-gap Structures in the Visible. *Physical Review Letters* **83**, 5274–5277 (1999). URL https://link.aps.org/doi/10.1103/PhysRevLett.83.5274.

[7] Adorf, C. S., Dodd, P. M., Ramasubramani, V. & Glotzer, S. C. Simple data and workflow management with the signac framework. *Computational Materials Science* **146**, 220–229 (2018). 1611.03543.
