# Supplementary material for: The diversity of three-dimensional photonic crystals
Source: Nat Commun. 2021 May 5;12:2543. doi: 10.1038/s41467-021-22809-6 (PMC8100142; doi:10.1038/s41467-021-22809-6)
Supplement: Supplementary file 2 — Description of Additional Supplementary Files [file 41467_2021_22809_MOESM2_ESM.pdf]

Description of Additional Supplementary Files

File Name: Supplementary Data 1

Description: This excel file contains two sheets corresponding to (1) all structures analyzed in this study and (2) structures found to support a photonic band gap in either their direct or inverse instantiation.

In (1) there are 1357 entries with 8 columns:

A. Space Group Number
B. Global Point Group
C. Local Point Groups
D. Pearson Symbol
E. Database Code – where “SIM” refers to “Found in simulation”, “ICSD” refers to the inorganic crystallographic structure database, and “COD” refers to the open crystallographic database. Numbers refer to the unique identifier for the corresponding database.
F. Direct – whether a PBG can be supported in the direct version of the structure
G. Inverse – whether a PBG can be supported in the inverse version of the structure
H. Structure DOI / URL – url for the original source of the structure, either a publication or database entry

In (2) there are 452 entries, each corresponding to a unique PBG found, and 10 columns:

A. Space Group
B. Pearson Symbol
C. Type – designates whether this entry corresponds to the “direct” or “inverse” instantiation o the structure
D. Gap above Band – band delineating the bottom of the PBG
E. max(Δω(ε=16)) – maximum band gap wise at ε=16
F. ω*(ε=16) – mid-gap frequency, in reduced units, of the band gap at ε=16 at the ratio of high and low dielectric constant which maximizes the band gap
G. min(ε_(Δω>0)) – minimum dielectric constant where a band gap is supported
H. Constituents – atomic species present in the natural analog, “_r” denotes an inverse structure
I. Database Code – where “SIM” refers to “Found in simulation”, “ICSD” refers to the inorganic crystallographic structure database, and “COD” refers to the open crystallographic database. Numbers refer to the unique identifier for the corresponding database.
J. Photonics DOI / URL – first instance of citing literature (to our knowledge) mentioning this structure or space group in the context of photonic band gaps
